# Optimizing Pluronic–PEI Nanocarriers for RNAi Delivery in Oral Cancer: From Polymer Synthesis to Functional Screening

**DOI:** 10.1021/acs.biomac.5c01011

**Published:** 2025-10-06

**Authors:** Cátia Domingues, Ivana Jarak, Jorge Coelho, Rui A. Carvalho, Francisco Veiga, Carla Vitorino, Marília Dourado, Ana Figueiras

**Affiliations:** † 37830 Univ Coimbra, Faculty of Pharmacy, Coimbra 3000-548, Portugal; ‡ REQUIMTE/LAQV, Drug Development and Technologies Laboratory, Faculty of Pharmacy, 37829 University of Coimbra, Coimbra 3000-548, Portugal; § Institute for Clinical and Biomedical Research (iCBR) Area of Environment Genetics and Oncobiology (CIMAGO), Faculty of Medicine, University of Coimbra, Coimbra 3000-548, Portugal; ∥ Department of Chemical Engineering, Rua Sílvio Lima- Pólo II, Univ Coimbra, CEMMPRE, Coimbra 3030-290, Portugal; ⊥ Department of Life Sciences, Faculty of Sciences and Technology, University of Coimbra, Coimbra 3000-456, Portugal; # Instituto de Investigação e Inovação em Saúde, University of Porto, Porto 4000-235, Portugal; ¶ Coimbra Chemistry Centre, Faculty of Sciences and Technology, University of Coimbra, Coimbra 3004-535, Portugal

## Abstract

Oral squamous cell carcinoma (OSCC) treatment is challenged by disrupted microRNA (miRNA) regulation, making efficient miRNA delivery essential. Here, we synthesized and screened Pluronic–polyethylenimine (Pluronic–PEI) nanocarriers for miRNA delivery in OSCC. Among several Pluronic variants, only L121 formed stable, fully cross-linked micellar nanogels with low-molecular-weight PEI (1.8 kDa), designated PP03. Its monomodal size, rough 3D morphology, and zeta potential > +30 mV provide colloidal stability and allow electrostatic miRNA complexation. The presence of a pH-sensitive ester linkage may enable endosomal escape through PEI’s proton sponge effect, combined with Pluronic-mediated osmotic modulation, which promotes the release of the therapeutic cargo at the site of action. These findings may lead to the PP03 outstanding profile in delivering miRNA100, which significantly reduced OSCC cell metabolic activity in 2D and 3D cultures and decreased spheroid size, particularly in highly metastatic models. Moreover, the noticeable mucoadhesion properties of PP03 and its hemocompatibility encourage its versatile application for oromucosal and intravenous administration. These results underscore the importance of polymer chemistry in developing functional miRNA nanocarriers to enhance oral cancer treatment.

## Graphical abstract

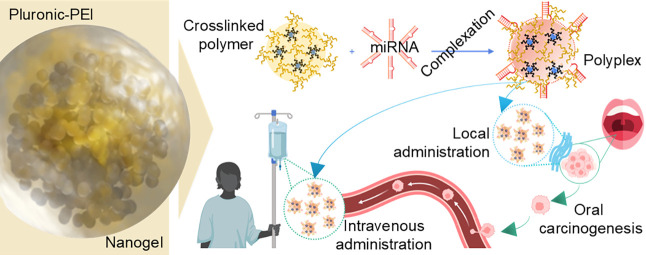

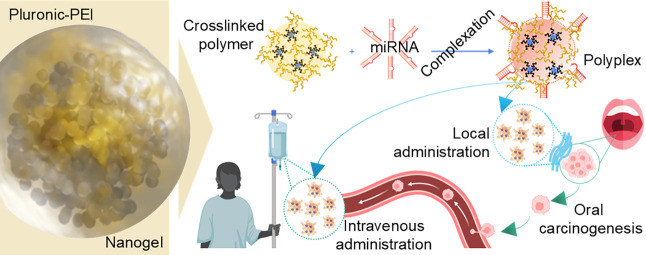

## 1 Introduction

The global oral cancer treatment market was anticipated to rise from USD 12.83 billion in 2024 to USD 22.2 billion by 2033, accompanied by a compound annual growth rate (CAGR) of 6.28% through the forecast period.^1^ Oral squamous cell carcinoma (OSCC) represents the most prevalent and clinically challenging form of oral cancer.^2^ The complex etiopathogenesis of OSCC is driven by multiple risk factors, including chronic tobacco and alcohol consumption, human papillomavirus (HPV) infection, and an aging population.^3^ Despite advances in diagnostic techniques, approximately 60% of OSCC patients are still diagnosed at a locally advanced stage, significantly limiting the effectiveness of standard treatments and contributing to a 5 year overall survival rate that remains below 50%.^4,5^

Considering these challenges, innovative therapeutic approaches are urgently needed. Among them, the targeted delivery of RNA interference (RNAi)-based agents has emerged as a promising strategy to modulate oncogenic pathways with high specificity.^6–8^ RNAi-based therapeutics, encompassing both small interfering RNAs (siRNAs) and microRNAs (miRNAs), mediate post-transcriptional gene silencing by binding to complementary mRNA (mRNA) sequences, leading to mRNA degradation or translational repression.^9^ Particularly, RNAi delivery to the oromucosal site and distant metastatic lesions offer a compelling route to overcome drug resistance, minimize systemic toxicity, and enhance therapeutic precision cancer treatment.^9,10^ However, challenges such as instability in circulation, poor cellular uptake, and off-target effects^11^ hinder their applicability in OSCC.

Since its introduction in 1995, polyethylenimine (PEI) has been extensively investigated as a nonviral gene delivery vector due to its high transfection efficiency, efficient cellular uptake, and relatively low immunogenicity compared to viral carriers.^12^ However, its clinical translation remains limited by significant drawbacks, including cytotoxicity, nonspecific biodistribution, and instability of PEI-nucleic acid polyplexes, all of which compromise intracellular delivery and reduce the therapeutic efficacy of nucleic acid-based treatments.^12,13^ To overcome these issues, various strategies have been developed to improve the safety and performance of PEI-based systems. One widely adopted approach involves the use of low-molecular-weight (LMW) PEI cross-linked with degradable linkers, including disulfide, ester, and amide bonds, which improves biocompatibility while maintaining transfection efficiency.^14–19^ Another promising strategy, originally reported by Kabanov et al., involves the incorporation of Pluronic block copolymers. These amphiphilic polymers not only enhance the in vitro and in vivo transfection efficiency of cationic vectors^20,21^ but also address safety concerns associated with the use of PEI.^22^ This approach has emerged from earlier work on cross-linking PEI with difunctionalized poly­(ethylene glycol) for antisense oligonucleotide delivery, which led to the introduction of the term “nanogels” in the field of polymer-based drug delivery systems by Kabanov et al.^21,23^ Although the applicability of different Pluronic–PEI combinations has been extensively studied in the delivery of pDNA,^24–26^ few studies report their application for RNAis complexation,^27–29^ particularly miRNAs. Actually, it was only in 2018 that Figueira’s group launched a set of original manuscripts reporting the study of Pluronic–PEI micellar nanogels for miRNA delivery in cancer therapy.^30–32^ However, the clinical application of polymer-based miRNA polyplexes in the treatment of OSCC remains an unmet medical need.

To explore the therapeutic potential of miRNAs in OSCC, we synthesized a library of Pluronic-based copolymers by conjugating various Pluronic derivatives with low molecular weight branched PEI (B-PEI) via a two-step synthesis, employing different hydroxyl group activators. The resulting polymers were thoroughly characterized for their structural, physicochemical, and mucoadhesive properties, focusing on interactions with mucin, which is a key component of the oromucosal environment. Their buffering capacity, miRNA complexation efficiency, cytocompatibility, and RNAi delivery potential were evaluated in the OSCC models. Finally, hsa-miR-100-3p mimics were employed to assess the transfection efficiency and anticancer activity of the lead formulation compared to B-PEI in 2D and 3D OSCC in vitro models, alongside toxicity testing in porcine tongue tissue and hemocompatibility assays.

## 2 Experimental Section

### 2.1 Synthesis of the Cross-Linked Polymers

A set of copolymers based on different Pluronics (F68, P105, L121, P123, and F127, Sigma-Aldrich, St. Louis, MO, USA) with a broad spectrum of molecular weights (MWs) and poly­(ethylene oxide) (PEO) and poly­(propylene oxide) (PPO) composition (Table 1) and LMW-branched-Polyethylenimine (B-PEI, 1.8 KDa, 40528, Alfa Aesor, Thermo Fisher Scientific, Haverhill, Massachusetts, EUA) were chemically conjugated using different multistep synthesis approaches, as previously described.^26,27,32,33^ Briefly, different Pluronics were activated with an excess of 1,1′-carbonyldiimidazole (CDI),^26,27,34,35^ acryloyl chloride,^32^ or succinic anhydride^36^ in different mol ratios. The reaction was carried out at temperatures ranging from 25 to 40 °C at least overnight. After that, the obtained products were purified by dialysis or precipitation with diethyl ether, and their structural characterization was performed by attenuated total reflectance Fourier-transform infrared spectroscopy (ATR-FTIR) and Proton Nuclear Magnetic resonance spectroscopy (^1^H NMR) to confirm the successful activation of the hydroxyl groups of the Pluronics. The intermediate activated Pluronics were then conjugated with different mole ratios (ranging from 1:1 to 1:10) of LMW-*b*-PEI for at least 48 h at different temperatures (from 25 to 50 °C). The resultant products were then purified by dialysis for at least 24 h, followed by lyophilization when applicable. The resulting Pluronic-PEI conjugates were designated as listed in Table 1.

### 2.2 Structural Characterization

Samples were structurally characterized by ^1^H NMR and ATR-FTIR, as detailed next.

#### 2.2.1 ATR-FTIR

ATR-FTIR was carried out using PerkinElmer Spectrum 400 dual mode FTIR/FT-NIR with a Universal ATR samplic accessory (PerkinElmer Spectrum). For this, samples were placed on the ZnSe crystal plate and scanned at 0.5 cm/s, with a resolution of 1–2 cm^–1^, between 4000 and 650 cm^–1^. A total of 16 to 32 scans were recorded.

#### 2.2.2 Proton Nuclear Magnetic Resonance Spectroscopy

^1^H NMR was carried out on a Bruker spectrometer (400, 500, or 600 Hz). For this, ca. 6 mg of each sample was dispersed in 600 μL of deuterated water (D_2_O) or deuterated chloroform (CDCl_3_), and the respective spectrum was acquired using the following parameters: sweep width of 7.2 kHz, radiofrequency pulse of 30°, with an acquisition time of 3 s, an interpulse delay of 10 s, and an average of 32 scans. Spectral analyses were performed using TopSpin (version 4.0.8, Bruker Biospin GmbH, Rheinstetten, Germany).

#### 2.2.3 Polymer Degradation Studies

Copolymer degradation was assessed by ^1^H NMR by observing the presence/absence of characteristic peaks in the regions δ 4.0–3.8 and δ 3.3–2.5 ppm characteristic of polymers bound by the ester moiety. For this, samples (150 mg) were dispersed in 100 μL of nuclease-free water and incubated at 37 °C under orbital shaking. 24 h later, the pH of each sample was adjusted to pH 5.0 or 7.0 using 1 M HCl or 1 M NaOH to mimic endosomal and cytoplasmatic compartments, respectively. After a further 48 h of incubation at 37 °C under orbital shaking, samples were collected and mixed with D_2_O (500 μL), and ^1^H NMR analysis was performed as described in Section 2.1.2. The same protocol was employed for polymer dispersions without a pH adjustment (control group).

### 2.3 Thermal Properties

#### 2.3.1 Thermogravimetric Analysis

The thermal stability of the samples was evaluated simultaneously by thermal analysis using a TG 209 F3 Tarsus (Netzsch, Germany) at temperatures from 25 to 600 °C, under a nitrogen atmosphere flow of 20 mL min^–1^ with a heating rate of 10 °C·min^–1^, using open alumina crucibles. The initial sample mass was about 16–17 mg. The percentage of mass loss was determined using Proteus Software (Netzsch).

#### 2.3.2 Differential Scanning Calorimetry

The thermal behavior of the samples was studied by differential scanning calorimetry (DSC) in a Netzsch DSC 204 F1 Phoenix model (Netzsch, Selb, Germany). The heat flow and the heat capacity were calibrated at 10 °C min^–1^ using, respectively, indium and sapphire standards. Samples weights ranging from 3 to 7 mg were placed in a concave aluminum pan with an ordinary closed aluminum lid. An empty pan was used as a reference. Pans were punctured and heated from −80 to 200 °C with a constant heating rate of 10 °C min^–1^ under a dry nitrogen purge flow of 20 mL min^–1^. Thermograms, the onset temperature (*T*_onset_), melting point (*T*_peak_), and enthalpy (Δ*H*) were recorded using Proteus version 8.0 Software (Netzsch, Germany).

### 2.4 X-ray Powder Diffraction

The crystalline behavior of the different samples was analyzed by X-ray powder diffraction (XRPD) in a MiniFlex 600 X-ray diffractometer (Rigaku, Tokyo, Japan). For this, samples were placed on a glass support and irradiated with CuKα radiation at 40 kV and 15 mA. The 2θ scan range was 3–40° with a step size of 0.01° and a scan speed of 5 s/°.

### 2.5 Determination of the Primary Amine Content

To quantify the B-PEI content in the synthesized Pluronic–PEI polymers, the primary amine content was estimated using the 2,4,6-trinitrobenzenesulfonic acid (TNBS) assay.^37^ A stock solution of B-PEI (1 mg·mL^–1^) was prepared in ultrapurified water (Ω = 18.2 MΩ·cm, TOC <1.5 μg/L; Sartorius, Göttingen, Germany) and serially diluted in 0.1 M borate buffer (pH 9.5) to generate a calibration curve (1–50 μg·mL^–1^, *n* = 3). Similarly, copolymer dispersions (1 mg·mL^–1^) were prepared in ultrapurified water and diluted in the same buffer to the desired concentrations. Then, 100 μL of each standard, sample, and ultrapurified water used as blank was transferred to a transparent 96-well plate in triplicate. A freshly prepared TNBS reagent (25 μL, 0.1% in borate buffer) was added to each well. The plate was incubated in the dark at room temperature for 30 min, and absorbance was recorded at 405 nm using a microplate reader (Synergy HT luminometer, BioTek, Winooski, VT, USA).^26^ The B-PEI content in the different synthesized Pluronic–PEI was estimated by interpolating from the average freshly prepared calibration curve obtained from 3 independent replicates.

### 2.6 Colloidal Properties Evaluation

Average hydrodynamic diameter and polydispersity index (PdI) were assessed by dynamic light scattering (DLS) and surface charge (zeta potential, ZP) by electrophoretic light scattering (ELS) using Zetasizer Nano ZS (Malvern Instruments, Malvern, UK). The hydrodynamic diameter was calculated at a backward scattering angle of 173° using the Stokes–Einstein equation, and ZP was determined according to the Henry–Smoluchowski equation. The measurements were carried out in triplicate at 25 and 37 °C using different solvents, as detailed in the Results section (Figure 4). Moreover, 1 mM KCl (filtered with 0.22 μm cellulose acetate syringe filters) was also used as a dispersant for the laser-Doppler anemometry measurements.

### 2.7 pH and Osmolality Assessment

The pH of the different tested samples was measured at room temperature using a digital pH meter (Consort C3010, Consort bvba, Turnhout, Belgium), calibrated with pH 4.00, 7.00, and 10.01 standard buffers. The pH electrode was directly immersed in several prepared samples, and pH was recorded after stabilization.

Osmolality was determined with a Vapor Pressure VAPRO 5520 (Wescor, Utah, USA). The osmometer was left to stabilize for 4 h and was calibrated with 100, 290, and 1000 mmol/kg standards before use. Osmolality was measured 80 s after 10 μL of samples was carefully placed on the appropriate paper disk.

### 2.8 Sterility

For sterility testing, samples were sterilized under aseptic conditions in a laminar flow cabinet using terminal filtration through 0.22 μm nylon membrane filters (13 mm; Labfil, Zhejiang, China). Both unfiltered and filtered dispersions were incubated at 37 °C for 30 min to allow for stabilization. Then, 10 μL of each sample was inoculated in Trypticase Soy Agar (TSA) plates (150 mm diameter) by using calibrated sterile plastic inoculation loops. The plates were incubated at 37 °C for 7 days. TSA alone served as a negative control, while *Staphylococcus aureus* (6538, American Type Culture Collection, Manassas, Virginia, United States) was used as a positive control. Microbial growth was assessed visually by the presence of colony-forming units.

### 2.9 Microscopy Studies for Polymer Characterization

Initially, microscopy studies were conducted using a Morphology 4 instrument (Malvern Panalytical, UK) equipped with various lighting models, including brightfield, diascopic, and episcopic LED lighting, as well as darkfield and episcopic lighting, unified with an 18 MP detector camera and a Nikon CFI 60 optical system. These attributes allowed for preliminary inspection of the size and shape of the selected synthesized polymers in bulk. For this, ca. 10 mg of the polymer in bulk was placed on a glass slide, and the morphological features were observed under a 50× magnification lens, allowing the detection and analysis of particles with sizes ranging from 0.5 to 50 μm.

The 3D morphological characteristics were observed by scanning electron microscopy (SEM). Before the analysis, the samples (in native structure or dispersed in water at 20 mg·mL^−1^) were properly spread on a double-sided carbon tap, mounted onto an aluminum stud, and dried under vacuum. Microphotographs were registered using a tungsten cathode scanning electron microscope JSM 6010LV/6010LA (Jeol, Tokyo, Japan), with an acceleration voltage of 10 kV.

Transmission electron microscopy (TEM) was also employed using a Tecnai G2 Spirit BioTWIN 100 kV TEM (FEI Company, Eindhoven). The freshly prepared samples were absorbed on copper grids covered with Formvar and dried for 5 min. Data was obtained by analysis 2.0 software.

### 2.10 Critical Micellar Concentration Assessment

The critical micellar concentration (CMC) was determined at 25 and 37 °C using the pyrene fluorescence method, following a previously adapted protocol.^38^ The intensity ratio of the first (*I*_1_) to third (*I*_3_) vibronic peaks in the pyrene emission spectrum, which indicates the polarity of the surrounding environment (i.e., the micellar core), was measured across a range of copolymer concentrations. The *I*_1_/*I*_3_ values were plotted against the logarithm of copolymer concentration, and the CMC was calculated by fitting the data to a Boltzmann sigmoidal equation.^39^

### 2.11 Determination of Buffer Capacity

The buffering capacity of the synthesized copolymers was evaluated by acid–base titration, normalizing the amount of B-PEI to 0.4 mg, as determined by the results obtained in the TNBS assay. 1 mM KCl was used as a dispersant and was also titrated.^40^ The digital pH meter was manipulated as detailed in Section 2.7. The pH of each sample was initially adjusted to ca. 12 using 1 M NaOH. Then, the pH was registered after adding 3 μL of 1 M HCl to each sample until the pH was reduced to ca. 2. Buffering capacity was calculated^41^ based on eq [Disp-formula eq1_1]

1.1 $$Buffering\;capacity\;\left(\%\right)=\left[\frac{\left(\Delta V\cdot C_{HCl}\cdot MW_{HCl}\cdot 10^{-3}\right)}{W}\right]\times 100$$

The pH versus the volume of 1 M HCl used to titrate each polymer was plotted, and a Boltzmann sigmoidal regression was performed. Δ*V* was obtained by the interpolation of each polymer curve for pH 7.5 and 4.5, respectively. *C*_HCl_ corresponds to the 1 M stock solution concentration of HCl used to titrate each sample, and MW_HCl_ corresponds to the molecular weight of HCl, which corresponds to ca. 36.5 g/mol. W, corresponds to 0.4 mg, which is the mass of B-PEI present in all tested samples.

### 2.12 Mucoadhesion Studies

The interaction between mucin (M2378, Sigma-Aldrich) and various polymers was studied using different in vitro methods, including turbidimetry,^42,43^ tensile strength analysis,^44^ and the assessment of colloidal properties.^43^

#### 2.12.1 Turbidimetric Titration

The mucin dispersions were prepared at 1 mg·mL^–1^ in ultrapurified water or in nonenzymatic artificial saliva pH 6.8 (composition: 5 mM NaHCO_3_, 7.36 mM NaCl, 20 mM KCl, 6.6 mM NaH_2_PO_4_·H_2_O, and 1.5 mM CaCl_2_ in ultrapurified water). The dispersions were stirred for 2 h, followed by 10 min of sonication and centrifugation at 170*g* for 5 min.^43^ Then, the supernatant was collected and preserved for further experiments. The synthesized polymers, B-PEI, and hyaluronic acid (HA, 14.8 kDa, Lifecore Biomedical LLC, Chaska, MN, USA) were dispersed in ultrapurified water at a final concentration of 1 mg·mL^–1^ for each experiment. LMW-Chitosan (Ch, 448869, Sigma-Aldrich) at 1 mg·mL^–1^ was prepared in 1% (*v*/*v*) acetic acid (33209, Honeywell, Germany). Ch and HA were used as positive and negative controls for mucoadhesion, respectively. After, different mass ratios of polymer/mucin dispersions, ranging from 0 to 1, were prepared by adding polymers to a fixed mass of mucin dispersion. Then, the mixtures of mucin and polymer dispersions were allowed to interact for 8 min at 37 °C. The turbidity of each sample (*n* = 3) was measured at 400 nm using a microplate reader (Synergy HT luminometer, Biotek, Winooski, VT, USA).

#### 2.12.2 Texture Analyzer

Mucoadhesion studies were also carried out using a texture analyzer TA.XT Plus (Stable Micro Systems Ltd., UK), equipped with an analytical probe (P/10, 10 mm Delrin), following an adapted protocol by Jones et al.^44^ Mucin disks with ca. 100 mg were prepared by direct compression using a hydraulic press (Speca Press., UK) with a 10 mm diameter die and at five tons compression force. Afterward, the mucin disks were visually inspected to ensure that no disintegration occurred and weighed to confirm that no mass was lost. Then, a mucin disk was attached to the analytical probe using double-sided adhesive tape, and the tested samples (200 μL) were placed in the mucoadhesion test rig apparatus (A/MUC) and maintained at 37 °C. The maximum detachment force (adhesiveness) and the work of adhesion (calculated from the area under the curve from the force–distance plot) were recorded by the Texture Exponent 6.1.16.0 software package (Stable Micro Systems, Surrey, UK). All of the measurements were conducted at least in triplicate.

#### 2.12.3 Colloidal Properties Assessment

Changes in the hydrodynamic diameter and PdI, as well as in the zeta potential of the mucin particles before and after the incubation with different polymers in a polymer/mucin (*w*/*w*) ratio ranging from 0 to 1 were registered as detailed in Section 2.6. The mucin and polymer dispersions were prepared as detailed in Section 2.12.1, using ultrapurified water or 1 mM KCl for the zeta potential measurements.

### 2.13 Polyplexes Preparation

Polyplexes were prepared by the complexation between the positive charges of the protonated amine groups (N) of the cationic polymers and the negative charges of the phosphate groups (P) of the double-stranded RNAi (simply abbreviated as RNAi and used in siRNA and miRNA calculations), as reported previously.^45^ Briefly, the volume of RNAi stock solution, *V*_RNAi_, was calculated as follows (eq [Disp-formula eq1_2]):

1.2 $$V_{RNAi}=\frac{\left(C_{RNAi,final}\cdot V_{final}\right)}{C_{RNAi,stock}}$$

where *C*_RNAi,final_ corresponds to the final concentration of RNAi in the sample (10–80 nM), *V*_final_ is the final sample volume (100 to 700 μL), and *C*_RNAi,stock_ = concentration of RNAi stock solution (2–100 μM).

Stock samples of the different Pluronic–PEI conjugates (PP01 to PP05) and the native B-PEI were prepared at concentrations ranging from 0.1 to 4.0 mg·mL^–1^, considering the B-PEI content determined by the TNBS assay (Table 1). Afterward, the volume of B-PEI required to prepare each polyplex formulation was calculated using the following eq [Disp-formula eq1_3]:

1.3 $$V_{PEI}=\frac{\left[\frac{\left[\frac{\left(C_{RNAi,final\;}\cdot MW_{RNAi}\right)}{\left(MW_{PO_{4}^{3-}}\cdot \phi_{N:P}\cdot V_{final}\right)}\right]}{\left(\frac{C_{PEI,stock}}{MW_{N}}\right)}\right]}{0.7}$$

where MW_RNAi_ = molecular weight of RNAi duplex (14,000), MW (PO_4_^3–^) molecular weight per phosphate group of RNAi (94.97 g·mol^–1^), Φ_N/P_ the molar ratio of B-PEI nitrogens to RNAi phosphates, *C*_PEI, stock_ = concentration of B-PEI in the stock polymer dispersion (normalized according to the % obtained in the TNBS assay), and MW_N_ = molecular weight per nitrogen of B-PEI (43 g·mol^–1^).^45^ Moreover, considering that at pH 7.4, ca. 70% of the amino groups of the PEI are protonated,^46–49^ 0.7 corresponds to the correction factor accounting for the concentration of protonated amino groups. Freshly prepared polyplexes were formed by adding the polymer to the RNAi for 5 s and incubating it at room temperature for at least 20 min to allow complexation between the positive charges of the cationic polymers and the negatively charged RNAi.

### 2.14 microRNA Selection

Initial miRNA selection was guided by our previously reported review^6^ and subsequently refined using a freely available online tool (https://www.mirnet.ca/Secure/MirNetView.xhtml, last access February 2025), which enables correlation of specific pathologies with implicated miRNAs. Squamous cell carcinoma of the tongue was chosen as the target disease, and five candidate miRNAs were identified from the resulting clusters. Corresponding mirVana miRNA Mimics (Homo sapiens hsa-miR-100-3p [MIMAT0004512], hsa-miR-127-3p [MIMAT0000446], hsa-miR-143-3p [MIMAT0000435], and hsa-miR-342-3p [MIMAT0000753]) were subsequently prescreened in vitro using a FuGENE SI Transfection Reagent–based kit to optimize transfection conditions. Following this prescreening, hsa-miR-100-3p (miRNA100 or simply miR 100) was selected for further studies. The mirVana miRNA Mimic Negative Control #1 (Catalog No. 4464058) was used as a scrambled negative control (miR −).

### 2.15 MicroRNA Complexation Efficiency

The ability of the different synthesized polymers and the native B-PEI to complex miRNAs was evaluated by the SYBRGold assay.^50,51^ Briefly, polyplexes were prepared to achieve N/P ratios ranging from 0.1 to 50, as described above, considering a fixed final miRNA content of 8 pmol/well in a 96-well plate. After 20 min, polyplexes were diluted in HEPES buffer (20 mM pH 7.4), and 100 μL of each preparation was distributed to the respective well of a 96-well black opaque plate. Then, 100 μL of SYBRGold (Invitrogen) stock solution prepared in water was distributed to each well, achieving a final dilution of 1:10,000, according to the manufacturing instructions. Fluorescence was measured using a BioTek Synergy HT microplate reader (Winooski, EUA) at 485/20 and 528/20 nm as excitation and emission wavelengths, respectively. The percentage of polymer-bound miRNA was calculated based on eq [Disp-formula eq1_4].

1.4 $$Binding\;affinity\;\left(\%\right)=100-\left[\left(\frac{\left(RFU_{\left(polyplex\right)}-RFU_{\left(free\;polymer\right)}\right)}{\left(RFU_{\left(free\;miRNA\right)}-RFU_{\left(nuclease-free\;water\right)}\right)}\right)\times 100\right]$$

where RFU is the relative fluorescence unit for the polyplexes at different ratios, free polymer corresponds to the polymer fluorescence when interacting with the dye used as a negative control, and free miRNA corresponds to the miRNA used to generate polyplexes (8 pmol/well of a 96-well plate, 112 ng, ensuring a final concentration of 80 nM) subtracted by nuclease-free water RFU used as a negative control. The sigmoidal binding curves were generated by plotting % of bound miRNA vs ratio (N/P), and the value at 50% (VC50) of the binding occurred was calculated.

### 2.16 Agarose Gel Retardation Assay

To evaluate the ability of the different synthesized polymers (PP01 to PP03) to package miRNA, the successful formation of polyplexes was confirmed by the agarose gel electrophoresis assay.^33^ For this, 1% (*w*/*v*) agarose gel in 1× Tris-acetate-EDTA (TAE) buffer containing 2.5 μL of SYBRGold was prepared. The synthesized polymers (PP01 to PP03), the native B-PEI, the miRNA100, and the respective polyplexes were prepared as reported above (Section 2.13) to achieve a final N/P ratio of 5.

### 2.17 Stability of the Polyplexes against Fetal Bovine Serum

The aggregation of polyplexes in the presence of serum was evaluated in terms of turbidity increase at 630 nm using a microplate reader BioTek (BioTek Instruments, Inc., Winooski, VT, USA). Polyplexes at the N/P ratios ranging from 2.5 to 10 were prepared as referred to in Section 2.13 considering a fixed final miRNA100 content of 8 pmol/well in a 96-well plate. Briefly, polymer and miRNA stocks were each diluted to 35 μL in HEPES buffer (20 mM, pH 7.4), combined by adding polymer to miRNA, vortexed for 5 s, and incubated for 20 min at room temperature to allow complexation. The mixtures were then supplemented with 280 μL of HEPES buffer containing 10% FBS (final volume 350 μL) and 100 μL was dispensed per well of a 96-well transparent plate in triplicate. The same protocol was applied for equivalent polymer concentrations as blanks, with miRNA replaced by an equal volume of nuclease-free water. Absorbance was recorded every 30 min over 240 min, and blank values were subtracted from the corresponding polymer–miRNA samples, and the results represent the absorbance after this correction, Abs = (Abs_polyplexes_ – Abs_polymer_).

### 2.18 In Vitro Studies Using Oral Cancer Cells

#### 2.18.1 Cell Lines and Culture

Two human immortalized OSCC cell lines, SCC-9 (CRL-1629, ATCC, Manassas, VA, USA) and HSC-3 (SCC193, Sigma-Aldrich, St. Louis, MO, USA, were utilized in this study to represent in situ and highly invasive phenotypes of OSCC, respectively. SCC-9 cells were cultured in Dulbecco’s Modified Eagle’s Medium-Nutrient Mixture F-12 (DMEM-F12) (Biowest, Nuaillé, France, L0093), supplemented with 400 ng/mL hydrocortisone (H0888, Sigma-Aldrich, St. Louis, MO, USA), 10% (*v/v*) fetal bovine serum (FBS, Biowest, Nuaillé, France), and 1% (*v/v*) of 100× antibiotic-antimycotic (Biowest, Nuaillé, France). HSC-3 cells were maintained in Dulbecco’s Modified Eagle’s Medium (DMEM) with high glucose (L0103, Biowest, Nuaillé, France). Both cell lines were kept in a humidified incubator at 37 °C with 5% CO_2_. According to the manufacturer’s guidelines, HSC-3 cells were used for up to 10 passages, while SCC-9 cells were cultured for a maximum of 20 passages. The cell lines were handled separately to prevent cross-contamination and misidentification and regularly tested for mycoplasma contamination.

#### 2.18.2 Cell Metabolic Activity

Following a previously established protocol, cell metabolic activity was assessed using the resazurin assay.^52^ Briefly, cells were seeded in 96-well plates at a density of 5 × 10^3^ cells per well (100 μL of a 5 × 10^4^ cells·mL^–1^ suspension) and allowed to adhere for 24 h. After this period, the medium was replaced with a fresh medium without or with the treatment protocols. Particularly, cells were exposed to increasing concentrations of native Pluronics, B-PEI, and Pluronic–PEI conjugates. For polyplex exposure, several preparation protocols were tested, with the selected method summarized in Figure 12A. Briefly, polyplexes were prepared at an N/P ratio of 5, aiming to test 80 nM miRNA per well (100 μL). Polymer and miRNA stock preparations (Section 2.13) were diluted separately to 35 μL in serum-free medium, then combined by adding the polymer to the miRNA, vortexed for 5 s, and incubated for 20 min at room temperature to allow for complexation. After, the mixture was supplemented with 280 μL of medium containing 10% FBS (final volume 350 μL), and 100 μL was dispensed per well, allowing for 3 replicates. Controls were prepared using the same protocol, with polymer and miRNA replaced by nuclease-free water. Cells were then incubated at 37 °C in a humidified atmosphere with 5% CO_2_ for 48 h. Subsequently, the medium was removed and replaced with fresh medium containing resazurin (R7017, Sigma) at a final concentration of 44 μM, followed by incubation for an additional 2–4 h. Absorbance was measured at 570 and 600 nm using a Synergy HT microplate reader (BioTek Instruments, Winooski, VT, USA). Cell metabolic activity was calculated as described in eq [Disp-formula eq1_5] and plotted against the Log_10_ of the polymer concentration. Half-maximal inhibitory concentrations (IC_50_) were determined using nonlinear sigmoidal regression analysis.

1.5 $$Cell\;metabolic\;activity\;\left(\%\right)=\left[\left(\frac{{\left(Abs_{\left(570nm\right)}-Abs_{\left(600nm\right)}\right)}_{treated\;cells}}{{\left(Abs_{\left(570nm\right)}-Abs_{\left(600nm\right)}\right)}_{untreated\;cells}}\right)\times 100\right]$$

#### 2.18.3 Cellular Uptake Analysis by Flow Cytometry

Oral cancer cells were seeded onto 12-well microplates (15 × 10^4^ cells/well) and incubated at 37 °C for 24 h. Subsequently, polyplexes were prepared at an N/P of 5 with scrambled Cy5-siRNA (SIC005-10 nmol, Sigma-Aldrich) in order to track cellular uptake (240 pmol of siRNA/well at the final concentration of 80 nM). Transfected cells were then washed twice with PBS to remove the remaining polyplexes from the cell surface, harvested, and centrifuged at 200*g* for 5 min. The pelleted cells were suspended with 300 μL of PBS pH 7.4 and allowed to recover for 30 min in a roller at 37 °C. Subsequently, cells were transferred to flow cytometry tubes, and the Cy5 mean fluorescence intensity was analyzed by flow cytometry using a six-parameter, four-color FACSCalibur flow cytometer (Becton Dickinson, San Jose, CA) by collecting at least 10,000 events in CellQuest software (Becton Dickinson, San Jose, CA). The results were analyzed using Paint-a-Gate software and expressed as percentage (%). The representative dot plots were obtained from a free web-based application for flow cytometry analysis, floreada.io.

#### 2.18.4 Coumarin-6 Uptake

Coumarin 6 (C6, 442631, Sigma-Aldrich) was employed as a model hydrophobic fluorescent dye to evaluate cellular uptake via fluorescence spectroscopy and imaging. A 2.8 mM C6 stock solution was prepared in absolute ethanol. Aliquots (10 μL) were transferred into Eppendorf tubes and allowed to evaporate under light-protected conditions in a fume hood for 30 min. Synthesized copolymer dispersions (1 mg·mL^–1^) were prepared in nuclease-free water. Then, 1 mL of each formulation or nuclease-free water (control) was added to the Eppendorf tubes containing dried C6 and stirred at 300 rpm overnight at 25 °C. Samples were then filtered under aseptic conditions using hydrophobic PTFE membrane filters to remove the unincorporated dye. The amount of encapsulated C6 was quantified by interpolating fluorescence values from the mean calibration curve generated in ethanol (0–21.5 μM; *y* = 3421.7*x* + 671.44, *R* ^2^ = 0.999, *n* = 3). Acetonitrile was used as the blank.

For cellular uptake studies, HSC-3 cells were seeded at 3 × 10^4^ cells/well in 96-well solid black plates (Santa Cruz Biotechnology, Dallas, TX, USA) and allowed to adhere for 24 h. Cells were then treated with free C6 or C6-loaded formulations, diluted in a complete culture medium (1:10), and incubated at 37 °C under a humidified 5% CO_2_ atmosphere. At selected time points (0.25, 0.5, 1, 2, 3, 4, 6, and 8 h), cells were gently washed three times with ice-cold PBS to remove noninternalized dye. To quantify internalized C6, 100 μL of acetonitrile was added to each well for dye extraction, and fluorescence was measured using a Synergy HT microplate reader (BioTek Instruments, Winooski, VT, USA) with excitation/emission wavelengths of 485/20 nm and 590/35 nm, respectively. All experiments were performed in triplicate, and results were expressed as the percentage of internalized C6 relative to initial fluorescence.

For qualitative analysis, HSC-3 cells were seeded at 3 × 10^5^ cells/well in 6-well plates for 24 h. Then, the medium was replaced by a freshly prepared one containing or not containing C6. Live-cell imaging was performed using a Carl Zeiss Axio Observer Z1 inverted fluorescence microscope equipped with a 10× objective. Five fields per condition were imaged using an AxioCam MR R3 camera, and images were processed using ImageJ software (NIH, USA).

#### 2.18.5 Acridine Orange Assay

The potential of endosomal escape of the different developed polyplexes, particularly those formed by PP03 or B-PEI with miRNA100, respectively coded as PP03miR100 and PEImiR100, was studied using an acridine orange-based microplate fluorescent assay with slight modifications.^53^ Briefly, HSC-3 cells were seeded at an initial density of 3 × 10^4^ in a 96-well solid black opaque plate (Santa Cruz Biotechnology, Dallas, Texas, USA) and allowed to attach for 24 h. Next, the medium was removed and replaced by a freshly prepared one with AO dye (3.77 mM stock solution prepared in 1 M HCl, 235474516, Sigma-Aldrich) at a final concentration of 14.1 μM in DMEM high glucose medium without phenol red (Capricorn Scientific, Ebsdorfergrund, Germany, DMEM-HXRXA) and incubated at 37 °C, 5% of CO_2_ for 15 min, protected from light. Then, cells were washed twice with PBS pH 7.4, and 100 μL of complete growth medium without phenol red was distributed for each well and the fluorescence was immediately acquired. Accordingly, the green fluorescence intensity was recorded at an excitation and emission wavelengths of 485/20 nm and 528/20, respectively, while the red fluorescence intensity was registered at an excitation and emission wavelengths of 530/25 and 590/35 nm, using the BioTek Synergy HT microplate reader (BioTek Instruments, Winooski, VT, United States). Wells containing 100 μL of medium without cells were used as background fluorescence controls. Afterward, the medium was removed and replenished, a group of cells were used as a control (without transfection), and another set of cells were subjected to the transfection protocol with PP03-miR100 and PEI-miR100. The plate was then moved to the microplate reader with controlled temperature at 37 °C, and the fluorescence was measured every 5 min for the initial 30 min and then at 60, 120, 180, and 210 min. The assay was performed in quadruplicate, and the results represent the ratio of red to green fluorescence for each tested condition at the respective acquisition time, normalized to the control (untreated cells).

A parallel set of 1.5 × 10^5^ HSC-3 cells/well were seeded in 12-well plates and treated as described above for analysis by fluorescence imaging of AO staining after 4 h of incubation without or with the polyplexes. After washing with PBS twice, the microscopic images were taken, and the green and red fluorescence were obtained using a Carl Zeiss Axio Observer Z1 fluorescent inverted microscope using the 10× magnification objective by capturing 5 images per test condition using an AxioCam MR R3 camera and processed using the ImageJ software.

#### 2.18.6 Homotypic HSC-3 Tumor 3D-Spheroid Study

The effect of PP03miR100 and PEImiR100 was tested on a 3D spheroid monoculture of the human metastatic HSC-3 cell line as a more predictable model of the disease, as previously reported by us with slight modifications.^52^ Briefly, 2.5 × 10^4^ HSC-3 cells (200 μL) were distributed into a 96-well black/clear round-bottom ultralow attachment spheroid microplate (Corning, New York, USA) and incubated for 4 days at 37 °C, 5% CO_2_ to form spheroids. A 50% media replenishment was performed on days 4, 7, 11, 14, and 18. Specifically, on day 4, after carefully removing 100 μL of the starting growing medium, 100 μL of fresh medium was administered, either without treatment (control) or containing 2× stock preparations of PP03miR100 or PEImiR100 at an N/P ratio of 5. At regular intervals, growth kinetics was evaluated up to 18 days after cell seeding. Over time, bright-field microscopy images of the morphology of the homotypic HSC-3 3D-spheroids were taken using the 5× objective magnification (Carl Zeiss Axio Observer Z1 microscope). The average diameter (three diameter measurements randomly evaluated in three different spheroids) was estimated using ImageJ. To guarantee that the diameter of spheroids on day 4, i.e., prior to treatment, follows a normal distribution, frequency plotting and quantile–quantile (Q–Q) plot were generated. On day 14, after acquiring bright-field microscopy images, 50% of the medium was substituted with resazurin (final concentration of 44 μM). The metabolic activity of spheroids was assessed as described in Section 2.18.2.

The surface morphology of the homotypic HSC-3 3D spheroids was examined by scanning electron microscopy using a tungsten cathode JEOL scanning electron microscope, model JSM 6010LV/6010LA (Tokyo, Japan). For this, at the end of the metabolic activity protocol, representative spheroids of the control and treatment groups were carefully collected and transferred to a double-sided carbon tape mounted onto an aluminum stud and allowed to dry for 5–10 min. The analysis was conducted at magnifications of 100× and 250× at an acceleration voltage of 1 kV.

### 2.19 Hemolysis Test

The capacity of the developed nanosystems to trigger hemolytic events was evaluated as a requirement to anticipate potential nanotoxicological adverse events.^54^ For this, fresh human blood obtained from anonymized healthy volunteers after written informed consent was collected in vacutainer tubes containing ethylene diamine tetraacetic acid (EDTA) as an anticoagulant. Blood was carefully and gently diluted in 0.9% (*w*/*v*) NaCl (B. Braun) to obtain a final blood concentration of 3.5% (*v*/*v*), followed by a reading step at 540 nm using a BioTek Synergy HT microplate reader (Biotek Instruments, Winooski, VT, United States) to guarantee the integrity of the erythrocytes. Formulations were prepared as 10× concentrates in HEPES buffer (20 mM, pH 7.4). Aliquots of 100 μL were gently transferred to 0.9 mL of diluted blood in Eppendorf tubes and incubated at 37 °C for 1 h under gentle orbital shaking (100 rpm, PSU-10i, Biosan). Controls included HEPES buffer (20 mM, pH 7.4; dispersant), 0.9% (*w*/*v*) NaCl (negative control, NC), and 4% (*v*/*v*) Triton X-100 (T8787, Sigma-Aldrich; positive control, PC). After the incubation period, the blood samples were centrifuged at 2655*g* for 10 min at 37 °C, 150 μL of the obtained supernatants was transferred to a transparent 96-well plate, and the absorbance of the hemoglobin released by the lysed erythrocytes was recorded at 540 nm using a BioTek Synergy HT microplate reader (Biotek Instruments, Winooski, VT, United States). The hemolytic activity was calculated as previously reported,^55^ using eq [Disp-formula eq1_6]:

1.6 $$Hemolyis\;\left(\%\right)=\left[\frac{\left(Abs_{sample}-Abs_{NC}\right)}{\left(Abs_{PC}-Abs_{NC}\right)}\right]\times 100$$

where Abs_sample_ denotes the sample absorbance, Abs_NC_ the negative control absorbance, and Abs_PC_ the positive control absorbance, each measured at 540 nm for the respective diluted blood samples.

### 2.20 HET-CAM Assay

The Hen’s Egg Test - Chorioallantoic Membrane (HET-CAM) assay was used as an alternative toxicological method to assess potential inflammatory or irritant properties. After 8 days of incubation of the fertilized White Leghorn hen eggs (50–60 g, Coren, San Cibrao das Viñas, Spain) in a climatic chamber at 37 °C and 60% RH, a small window of about 1 cm^2^ was made in the eggshell to expose the CAM. Then, 200 μL of each formulation was carefully administered on the CAM. NaCl (0.9%) and NaOH (0.1 N) solutions were used as negative and positive controls, respectively. CAM vessels were visually monitored for 5 min to assess the appearance of hemorrhage, vascular lysis, coagulation, hyperemia, or other changes in the CAM vessels.

### 2.21 Ex Vivo Metabolic Activity Studies in Healthy Tongue Tissue

Porcine tongue epithelium was used due to its high similarity to the human mucosa.^56^ The impact of the developed formulations in normal tissue was assessed by an optimized protocol described by us previously.^52^

### 2.22 Statistical Analysis

Data were presented as mean ± standard error of mean (SEM) of at least three independent experiments. The statistical analysis was performed via GraphPad Prism 8.0.1 software (San Diego, CA, USA) as specified per each figure caption. In brief, statistical significance of the model fittings and regression terms was assessed using ANOVA (*p* value < 0.05) and Student’s *t*-test (95% level of confidence, α = 0.05). Non-significant coefficients were removed from the model via backward selection. Differences among test groups were evaluated using one-way ANOVA followed by Tukey’s, Dunnett’s, or Sidak’s multiple comparison tests (*p* < 0.05).

## 3 Results

### 3.1 Characterization of the Synthesized Polymers and B-PEI Composition

Different synthetic approaches were used to conjugate B-PEI with different Pluronics (Figure 1). Table 1 presents a summary of the compositions of native Pluronics and the resultant conjugation products used in this study.

**Figure 1:**
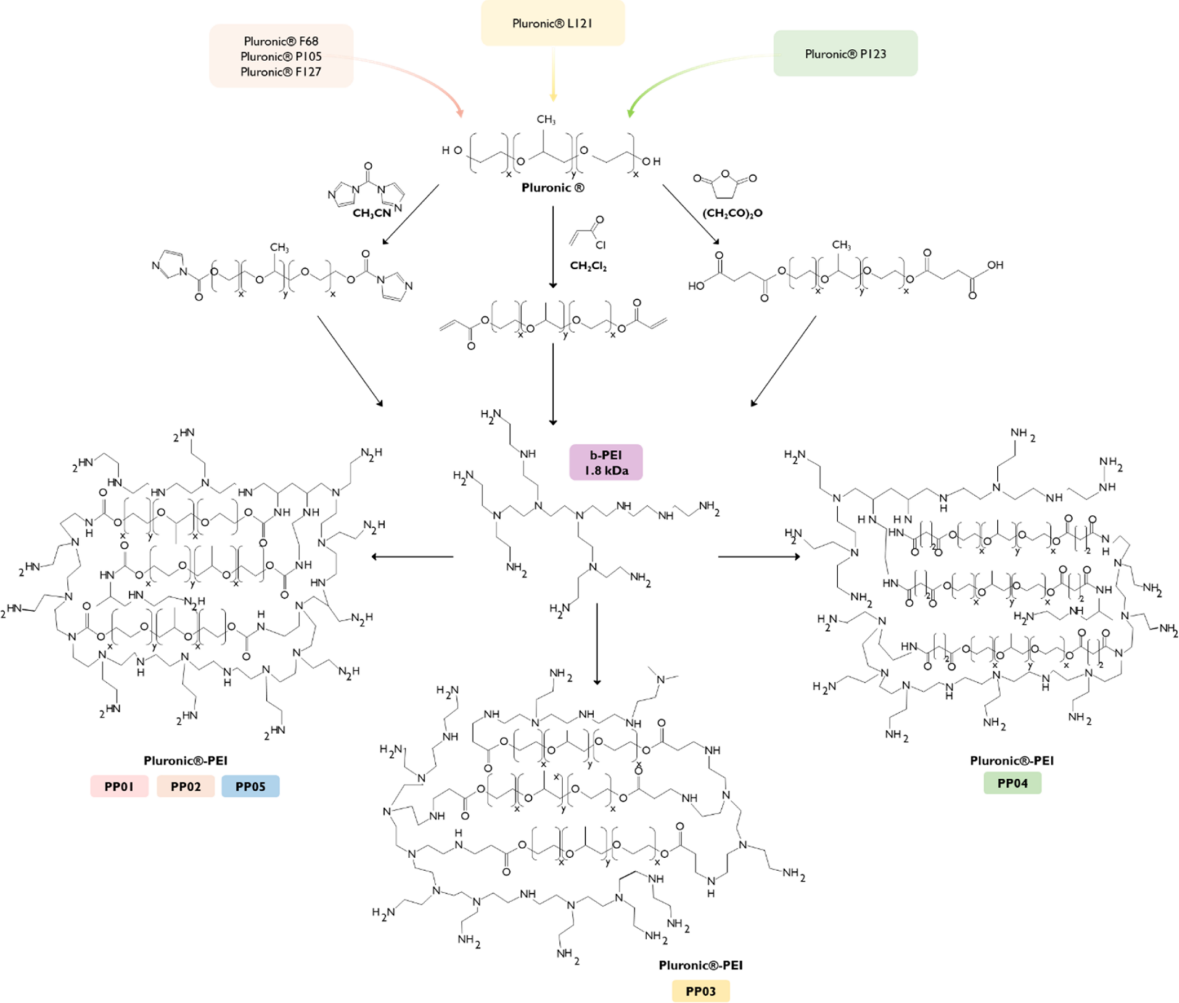
Schematic representation of the chemical reaction strategies employed to obtain the Pluronic–PEI conjugates.

**Table 1:** Structural Characteristics of Native Pluronics and the Resultant Conjugates with LMW-B-PEI = 1.8 kDa, Respectively

pluronic	poloxamer	MW (kDa)^a^ and^b^	total average PEO units^a^	total average PPO units^a^	HLB^b^	conjugate products code	ratio pluronic/PEI (estimated by ^1^H NMR)	% of PEI (estimated by the TNBS assay)
F68	P188	8.40	152.7	28.97	>24	PP01	1:0.70	12 ± 0.27
P105	P335	6.50	73.86	56.03	12–18	PP02	1:0.42	10 ± 0.20
L121	P401	4.40	10.00	68.28	1–7	PP03	1:2.0	42 ± 2.0
P123	P403	5.75	39.20	69.40	7–12	PP04	1:0.16	4.4 ± 0.29
F127	P407	12.6	200.4	65.17	18–23	PP05	I: 2.9	18 ± 0.11

a Ref 57.

b Ref 58.

The structural characterization of the native Pluronics and B-PEI, as well as the activated Pluronics and Pluronic–PEI conjugates was performed using ^1^H NMR (Figure 2) and FTIR (Figure 3). Generally, native Pluronics present a multiplet around δ 1.1 ppm, which is attributed to the protons of the PPO CH_3_ groups, the broad peaks from about δ 3.65 to 3.45 ppm belong to the PPO CH_2_ protons, and the sharp singlet at δ 3.7 ppm belongs to the PEO CH_2_ protons.^59^ In the IR spectrum, the native Pluronics displayed characteristic absorption bands at 1058 cm^–1^, indicative of the C–O stretching vibration of primary alcohols, and at 842 cm^–1^, 1100 cm^–1^, and 1342 cm^–1^, which are ascribed to the symmetric, asymmetric, and undefined stretching modes of the C–O–C linkage within the polymer backbone, respectively. Furthermore, the peaks at 1466 cm^–1^ and 2881 cm^–1^ are attributed to the vibrational modes of the CH_3_ groups.^60,61^ In Pluronics activated with CDI, such as F68 and P105, the reaction resulted in partial activation of the hydroxy groups, as demonstrated by ^1^H NMR spectroscopy (Figure 2). The formation of the conjugated Pluronic-CDI was confirmed by the appearance of typical imidazole multiplets in the aromatic region (δ 7.14, 7.67, and 8.4 ppm) and a multiple corresponding to PEO CH_2_ groups in the α position of the carboxyl group (δ 4.37 ppm) (Figure 2). These results were in line with those obtained by FTIR, as the appearance of an additional band at ca. 1760 cm^–1^ typical of C=O stretching indicated the presence of a carbonyl group, as previously described for other CDI-activated Pluronics (Figure 3).^26^ Subsequent nucleophilic substitution with B-PEI, resulting in the coded PP01, PP02, and PP05, respectively, was performed. In the ^1^H NMR spectrum, broad multiplets appeared between δ 2.6 and 3.1 ppm, along with a downfield shift of the CH_2_ protons adjacent to the carbonyl group (δ 4.25 ppm), indicating the possible successful conjugation of B-PEI to the synthesized Pluronic-CDI polymers (Figure 2). In an attempt to understand the composition of the conjugates, the molar ratio between Pluronics and B-PEI was determined by integrating the PPO methyl proton peaks (δ 1.1 ppm) and the B-PEI proton peaks (CH_2_–CH_2_–NH–, δ 2.7–3.4 ppm). This analysis yielded molar ratios of 1:0.7 (13.1% of B-PEI) for PP01, 1:0.42 (10.3% B-PEI) for PP02, and 1:2.9 (29.3 % of B-PEI) for PP05. These results are in good agreement with the TNBS assay, which estimated the B-PEI content to be approximately 12% in PP01, 10% in PP02, and 18% in PP05. ATR-FTIR further analyzed the resultant Pluronic–PEI to understand if characteristic bands of the polyurethane cross-linked polymers were present. As can be observed in Figure 3, the presence of bands at ca. 3350 cm^–1^ could be ascribed to the stretching vibration of the –NH group in the urethane linkage (−NH–C­(=O)–O−). However, upon a tailored ATR-FTIR protocol to identify typical bands present in polyurethane cross-linked polymers, it was not possible to identify typical bands attributed to urethane linkage, particularly, 1653 cm^–1^ (C–O),^62,63^ 1500 cm^–1^ (N–H and N–C) or 1224 cm^–1^ (C–O), leaving some doubts regarding the degree of cross-linking of this set of CDI-based activated Pluronics–PEI. Earlier studies using CDI as an activating agent demonstrated incomplete activation of Pluronics even with the excess of the activating agent.^26,64^ Consequently, a mixture of products can be isolated, including free, unmodified Pluronic. These results were also corroborated by the presence of characteristic crystalline melting temperatures (*T*_m_) closer to the native Pluronics, indicating the presence of semicrystalline regions in the synthesized polymers PP 01, 02, and 05 (data not shown), which may indicate the possible lack of complete or extensive cross-linking with B-PEI.^60,65^ Interestingly, the resulting polymers exhibited greater thermal stability than the starting materials, particularly B-PEI, as indicated by the thermoanalytical curves (data not shown). These results may designate the formation of derived products with enhanced thermal properties,^65^ which, together with prior evidence that unmodified Pluronic stabilizes Pluronic–PEI polyplexes and enhances transfection efficiency,^64,66^ supports a more comprehensive characterization of the newly synthesized products.

**Figure 2:**
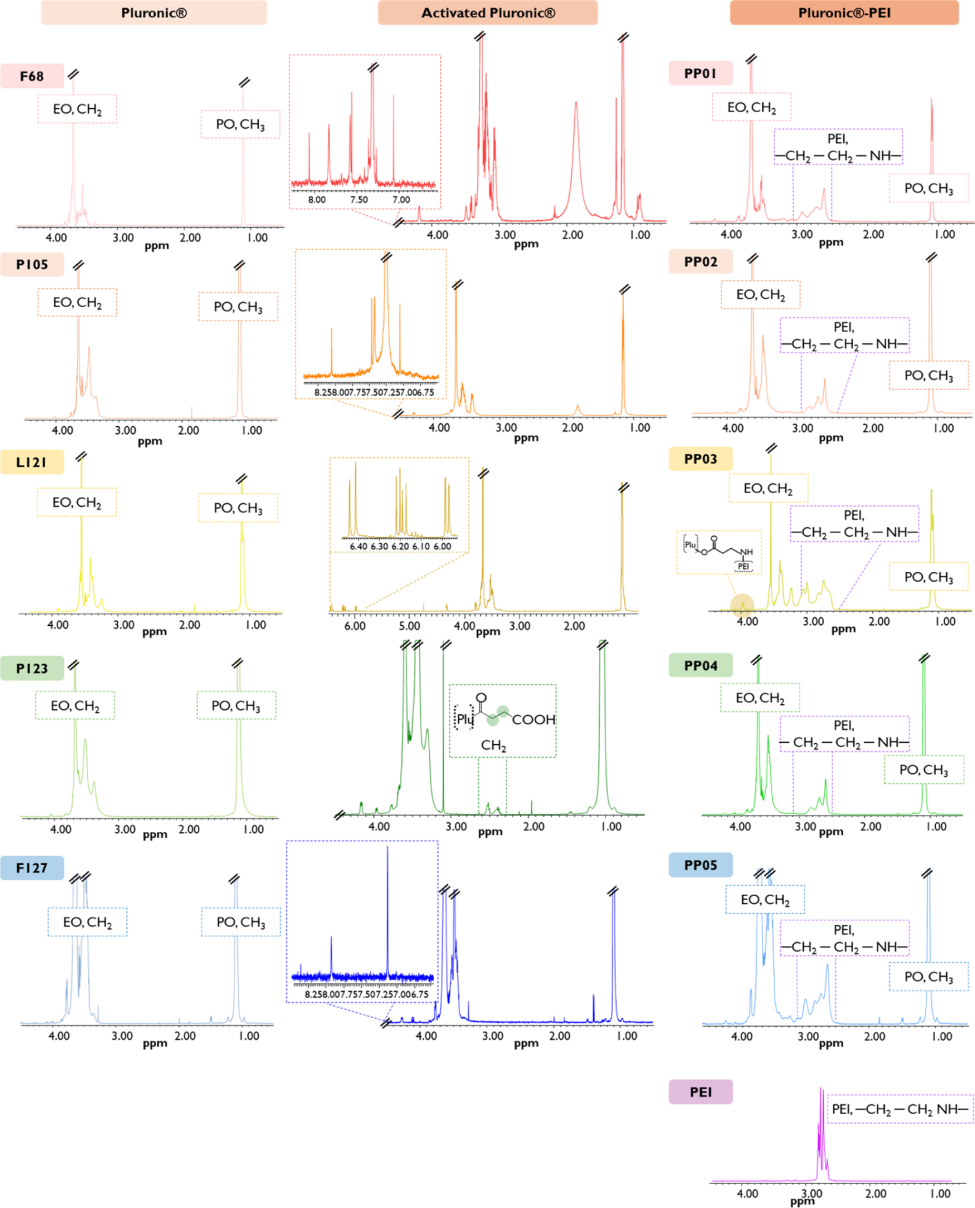
^1^H NMR spectra of native Pluronics (F68, P105, L121, P23, and F127), B-PEI (PEI), activated Pluronic intermediates, and the corresponding Pluronic–PEI conjugates (PP01, PP02, PP03, PP04, and PP05).

**Figure 3:**
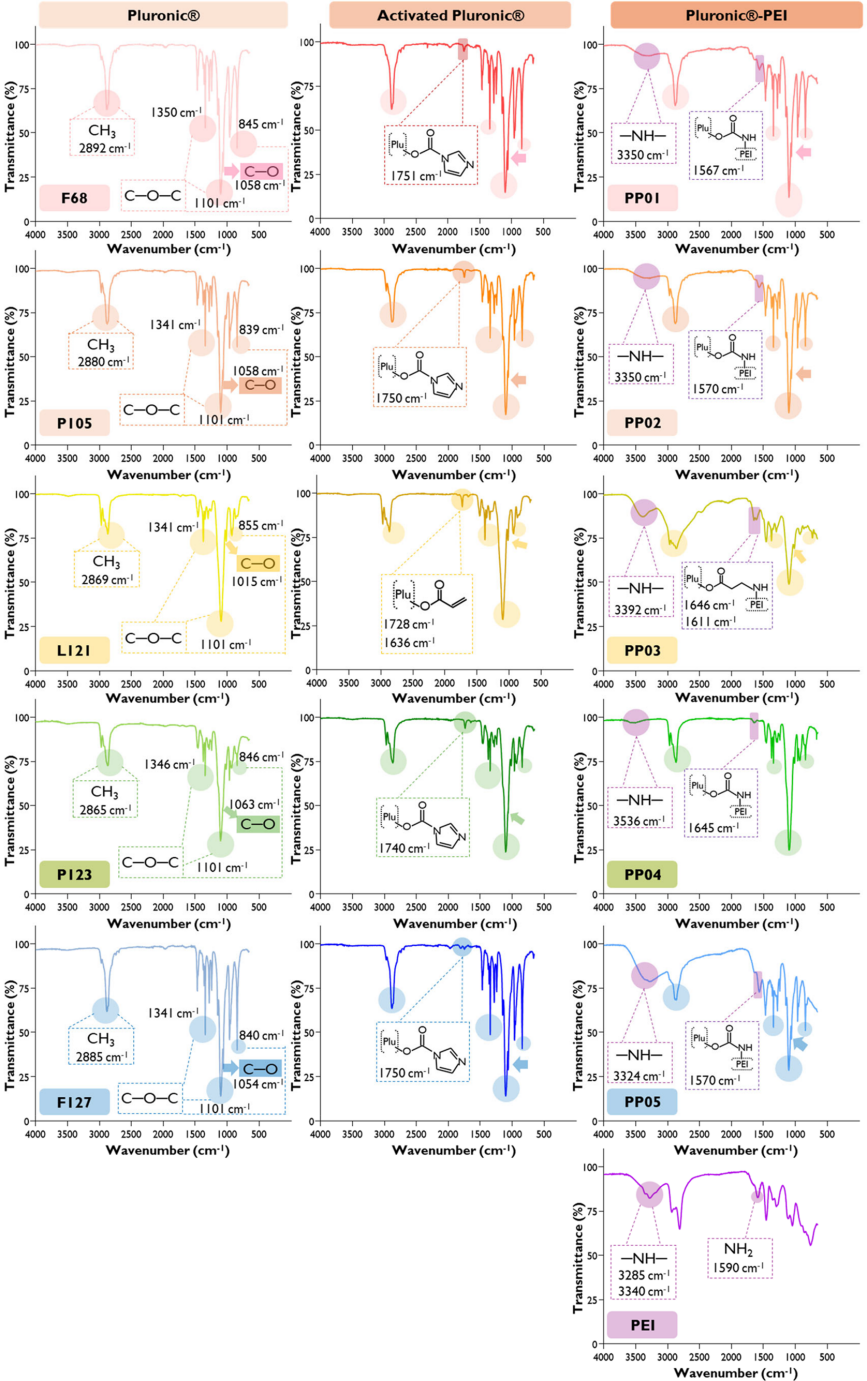
ATR-FTIR spectra of native Pluronics (F68, P105, L121, P23, and F127), B-PEI (PEI), activated Pluronic intermediates, and the corresponding Pluronic–PEI conjugates (PP01, PP02, PP03, PP04, and PP05).

On the other hand, when acryloyl chloride was used to activate Pluronic L121, hydroxyl groups were fully activated (Figure 2), particularly delineated by the appearance of characteristic peaks of vinyl groups in the region of δ 5.97–6.45 ppm, indicating the presence of diacrylate groups in the activated Pluronic. This result was also corroborated by FTIR analysis, where a band representative of the stretching of the C=O ester bond appeared, confirming the successful activation of Pluronic. The resulting Pluronic L121 diacrylate was subsequently grafted with B-PEI, yielding PP03 with approximately 42% B-PEI as determined by the TNBS assay (Table 1). This value is in reasonable agreement with the ^1^H NMR analysis, which indicated a 1:2 molar ratio between the activated Pluronic L121 and B-PEI (Figure 2), corresponding to approximately 46% B-PEI in PP03. The successful conjugation of PP03 was further validated by its IR spectrum, which displayed new characteristic B-PEI bands in the synthesized polymer, particularly between 3200 and 3400 cm^–1^, corresponding to the stretching vibrations of the secondary amine groups (-NH-) of B-PEI.^33^ Moreover, in PP03, only one thermal event, namely, the glass transition, was observed (Figure S1), suggesting the presence of extensive cross-linked nature of this synthetic polymer. The glass transition temperature (*T*_g_) of PP03 is higher than that of the native non-cross-linked polymers, which may indicate its complete cure and successful cross-linking. In fact, the higher cross-linking density also restricts polymer chain mobility, which has been implicated in the increase of *T*_g_.^67,68^ Concomitantly, the cross-linked polymer PP03 was found to be more stable than the starting material, namely, B-PEI (Figure S2). A weight loss between 40 and 155 °C was observed, which may be indicative of the presence of residual moisture or solvents that were unable to volatilize from the cross-linked network.^69^ This was followed by additional weight loss steps. The main weight loss detected at 354 °C (45.9%) could be attributed to the existence of a higher density of –OH groups near the ester linkages that can contribute to an acceleration of the thermal degradation of PP03, as observed in the thermal degradation of other polyacrylates.^70,71^ Moreover, as can be observed in the X-ray analysis (Figure S3), PP03 demonstrated an amorphous profile, corroborating its complete cure.^72^

In the case of Pluronic P123, the activation of the –OH groups was achieved in a reaction with succinic anhydride. In the ^1^H NMR spectrum, the appearance of multiplets at δ 2.52 and 2.41 ppm, which are characteristic of the –CH_2_ groups of succinic esters, indicated the successful activation of Pluronic P123. The activated P123 was subsequently conjugated with B-PEI, resulting in PP04 with a molar ratio of 1:0.16 (4.8% B-PEI) as determined by ^1^H NMR, which is in accordance with the B-PEI content of approximately 5% estimated by the TNBS assay (Table 1). These results, together with the thermal analysis, may indicate a limited cross-linked density of PP04, as no complete cure was observed, and the presence of semi-crystalline domains characteristic of the native P123 remained (data not shown). Even though the disappearance of the 1750 cm^–1^ characteristic band of carbonyl groups of ester moieties in the IR spectrum of the PP04 (Figure 3) may possibly suggest the formation of a conjugated polymer.

At this stage, a broad range of Pluronics, differing in PPO/PEO ratios, molecular weights, and related physicochemical properties such as hydrophilic–lipophilic balance (HBL) and CMC, were conjugated with various PEIs characterized by distinct degrees of branching and molecular weights. However, the use of different linkers, polymer feeding ratios, and experimental conditions (solvent, temperature, polymer concentration, and reaction time) often resulted in a variety of final cationic copolymers with varying degrees of polymerization, even when the same Pluronic/PEI combination was used.^29^ Indeed, despite the high hydrophilicity of Pluronic F68 (HBL = 29) and the reaction conditions may anticipate the F68–PEI reaction stoichiometry, the activation of F68 with bis­(trichloromethyl)­carbonate/*N*-hydroxysuccinimide followed by reaction with an excess of 2 kDa B-PEI produced a conjugate in which several Pluronic molecules were found per one molecule of PEI.^73^ Conjugates with a 1:1 ratio of Pluronic to 2 kDa B-PEI have also been observed with another hydrophilic Pluronic, F38 (HBL = 31), where the high CMC and the presence of non-aggregated, activated Pluronic chains under the reaction conditions favor the formation of conjugates with this specific stoichiometry.^26,64^ On the contrary, the proximity of multiple activated PEO chains in micelles formed by Pluronics with lower CMC might promote the conjugation of multiple Pluronics with the same PEI molecule.^74^ Nonetheless, depending on the reaction conditions, other types of high-molecular-weight products based on the cross-linking of polymer chains have also been described.^29^

Although Pluronic–PEI systems have been extensively investigated as pDNA transfecting agents, there are fewer published reports about their efficacy in the delivery of RNAi.^27,28,75^ Even though these types of transfecting agents seem to be efficient in delivering large nucleic acids with an abundant number of negatively charged groups, the structural factors that might govern the delivery of different RNAi are less well-known. Recently, our group has demonstrated the successful transfection of miRNA-145 and miRNA-29b in osteosarcoma and non-small cell lung cancer cells using L-64- and P103-PEI conjugates, respectively.^31,33^ In the latter case, comparison of transfection efficacy indicated a possible influence of the polymer composition on the structural parameters of the polyplexes and the delivery of therapeutic cargo.

Therefore, as a continuation of our efforts to understand the influence of Pluronic–PEI architecture on RNAi delivery, particularly miRNAs, the B-PEI derivatives developed in this study (PP01, PP02, PP03, PP04, and PP05) were further evaluated by assessing their colloidal and physicochemical properties, as well as their stability in forming RNAi complexes, as described in the following sections.

### 3.2 Colloidal Properties of Synthesized Polymers

The colloidal properties of the synthesized polymers were evaluated in terms of hydrodynamic diameter, PdI, and zeta potential and were used to evaluate stability after dilution, upon filtration, at different temperatures (25 and 37 °C) and in different media (nuclease-free water and HEPES buffer, pH 7.4) (Figure 4). In general, the observed parameters depend significantly on the tested conditions and the type of polymer. Across all the tested polymers and conditions, PP04 presented the most uniform characteristics. Among the tested samples, PP04 showed a small size (20–30 nm, Figure 4A) and low PdI (Figure 4B), but its minimal positive zeta potential (Figure 4C), reflecting low cationic PEI content, may hinder effective complexation and delivery of short-chain RNA. It was also possible to observe that the dilution of the developed synthesized polymers impacts their colloidal properties, particularly that of PP03 which may need extra time to stabilize after being diluted. In fact, after filtration at 25 °C, the dispersed polymers seem to undergo size reduction due to the removal of large aggregates, although the PdI values for PP01 and PP02 remain higher than 0.5. Moreover, it is interesting to note that the zeta potential remains similar, except for PP01. Under conditions that mimic physiological temperature (37 °C), the size of the filtered formulations has remained similar to those at 25 °C, accompanied by relatively stable positive zeta potential and reduction in the PdI values (PP01 and PP02). To test the impact of more complex physiological conditions, HEPES buffer (20 mM, pH 7.4) was used at 37 °C. Although the size of the dispersed polymers has remained similar to that observed in nuclease-free water at the same temperature, in most cases, an increase in PdI was observed (PP01, PP02, and PP04). Furthermore, the zeta potential continued positive for all the synthesized polymers in a function of the measured pH. As the zeta potential is influenced by ionic strength, the use of 1 mM KCl was considered. The nature of the media mimicking physiological conditions (HEPES and KCl at pH 7.4 and 37 °C) has strong influence on zeta potential across the tested polymers and interacts strongly with physicochemical and structural parameters of synthetic polymers. In the case of KCl, the inversion of zeta potential was observed when PP01 and PP04 were prepared at 37 °C, and the positive surface charge was lost. A similar effect was observed for B-PEI prepared in nuclease-free water at 10 mg·mL^–1^ (25 °C), 1 mg·mL^–1^ (37 °C), or in KCl. The charge of B-PEI remains positive (ca. 10 mV) when HEPES buffer pH 7.4 was used as a dispersant. In the case of B-PEI, the observed trend in zeta potential values seems to correlate with the pH values of solutions in different media. Due to the p*K*_a_ values of different amino groups, the protonation of individual amines could eventually decrease and remain very low in the cases of high pH (pH > 10). Overall, among the synthesized polymers, PP03 presented the most interesting profile across all tested conditions, with a hydrodynamic diameter of ca. 200 nm with low fluctuations in PdI. In fact, in nuclease-free water, a uniform size distribution was observed with a minor secondary peak above 1000 nm, which was occasionally observed in DLS analyses, consistent with a small fraction of transient aggregates (Figure S4A–C).^76^ However, PP03 under biologically relevant conditions (HEPES buffer, 37 °C) showed predominantly monomodal intensity distribution (Figure S4D), with low PdI values and a zeta potential higher than +30 mV, indicating not only colloidal stability but also an interesting surface charge profile to connect RNAi molecules electrostatically.

**Figure 4:**
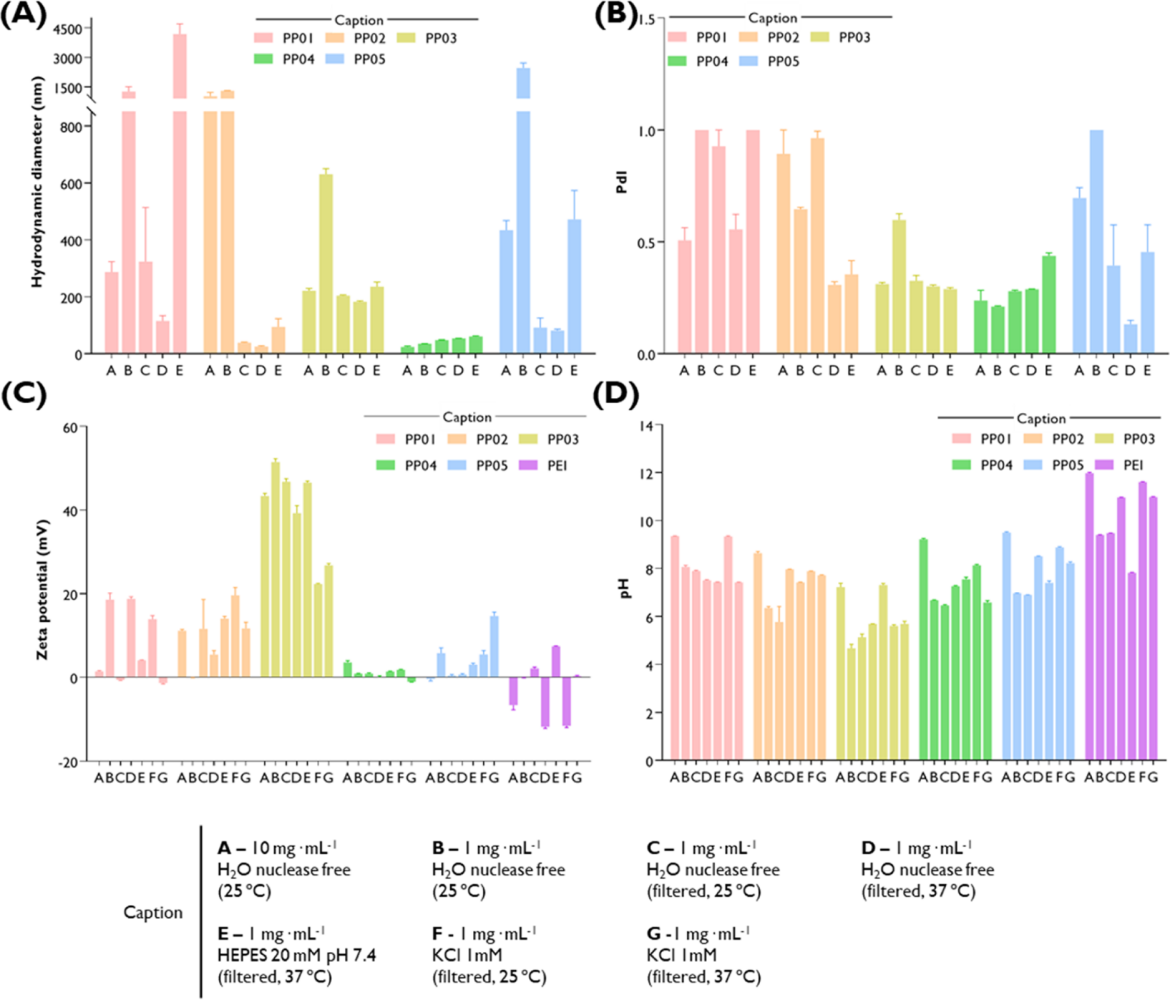
Colloidal properties of the Pluronic–PEI conjugates (PP01 to PP05) and the respective pH. (A) Hydrodynamic diameter (nm), (B) polydispersity index (PdI), (C) zeta potential (mV), and (D) pH of the formulations. The formulations were prepared at various concentrations and dispersed in different media, and their properties were monitored at different temperatures, as detailed in the caption.

### 3.3 Buffer Capacity, miRNA Complexation, and Stability in Serum

The developed polymers were evaluated in terms of their buffer capacity, an important parameter regarding the possible in vitro translation and efficacy to deliver cargos and promote endosomal escape.^12^ The results revealed that the synthesized polymers present values of buffer capacity higher than or near that of B-PEI (ca. 26.7%) in the pH range of 7.5–4.5 (Figure 5A).

**Figure 5:**
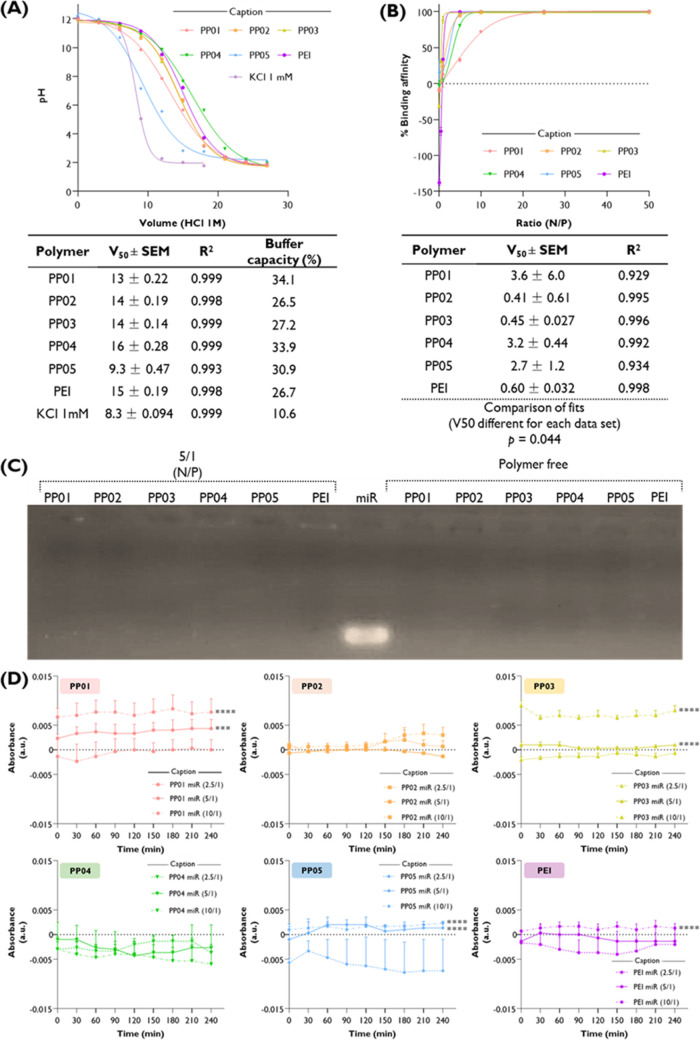
Buffer capacity, miRNA complexation, and stability in serum of the synthesized polymers (PP01 to PP05) and the native B-PEI (PEI). (A) Buffer capacity was determined by plotting pH against the cumulative volume of 1 M HCl added to each sample. The midpoint volume (V50) was obtained, and the model fit was confirmed by high R^2^ values (>0.99). The percentage (%) of buffer capacity was calculated as described in Section 2.11 over the pH range 7.5–4.5. (B) Binding affinity at increasing N/P ratios was evaluated, and the 50% binding point (V50) was derived from the corresponding curves, which showed good model fits based on the R^2^ values. (C) Stability of Pluronic–PEI/miRNA polyplexes at N/P ratios 2.5–10 was assessed in HEPES buffer (20 mM, pH 7.4) containing 10% FBS. Turbidity was monitored by plotting corrected absorbance values (Abs = Abs_polyplexes_ – Abs_polymer_) every 30 min during 240 min. The results represent the mean ± SEM of *n* ≥ 3 experiments. Note that some error bars are too small to be shown. Significant differences were defined as follows: ***p < 0.001 and ****p < 0.0001 (compared to the N/P ratio of 2.5). Note that some error bars are too small to be shown.

To investigate the binding affinity between the synthesized polymers and double-strand miRNA, polyplexes with different N/P values were prepared and compared with B-PEI (Figure 5B). The 50% binding affinity was reached for the ratio of 0.6 ± 0.03 in the case of B-PEI, while the same binding affinity for PP02 and PP03 was observed at a ratio of 0.41 and 0.45 (*p* < 0.005), respectively, demonstrating the outperforming binding capacity for the miRNA. These results are in agreement with the gel electrophoresis study (Figure 5C) that revealed that complete complexation of miRNA occurred at an N/P ratio of 5. The stability of transfecting agents and their complexes is one of the mandatory prerequisites for successful transfection. Therefore, the stability of polyplexes in HEPES buffer with 10% FBS was evaluated, revealing that stability depended on both the polymer type and the N/P ratio (Figure 5D). It is particularly relevant to emphasize that under physiological-like conditions, in the polyplexes prepared at low N/P (2.5–10). there was almost no impact on the stability of the nanosystems. Turbidimetry impact is close to 0 for almost all the tested polyplexes, except for those prepared based on PP01. Hence, considering the overall results summarized in Figure 5, polyplexes prepared at an N/P ratio of 5 were selected for further investigation.

Therefore, the colloidal properties of the Pluronic–PEI conjugates (PP01 to PP05) and the native B-PEI (PEI) complexed with miRNA100 at an N/P ratio of 5 were evaluated in HEPES buffer (20 mM, pH 7.4) at 37 °C and compared with the corresponding empty polymeric formulations (Figure 6). The results revealed that the hydrodynamic diameter (Figure 6A), the PdI (Figure 6B), and the zeta potential (Figure 6C) were impacted in a polymer-dependent manner. Moreover, the complexation of the polymers with the miRNA generally seemed to stabilize the different developed formulations, resulting in a transversal decrease in hydrodynamic diameter and PdI. Notably, PP03 maintained a hydrodynamic diameter of ∼200 nm after complexation, with no significant change in PdI and no evidence of new peaks or aggregation, as shown by the intensity-based particle size distribution curves (Figure S5). This stability upon nucleic acid loading is consistent with previously obtained results by our group, where minimal changes in size and PdI have been documented following miRNA complexation.^33^

**Figure 6:**
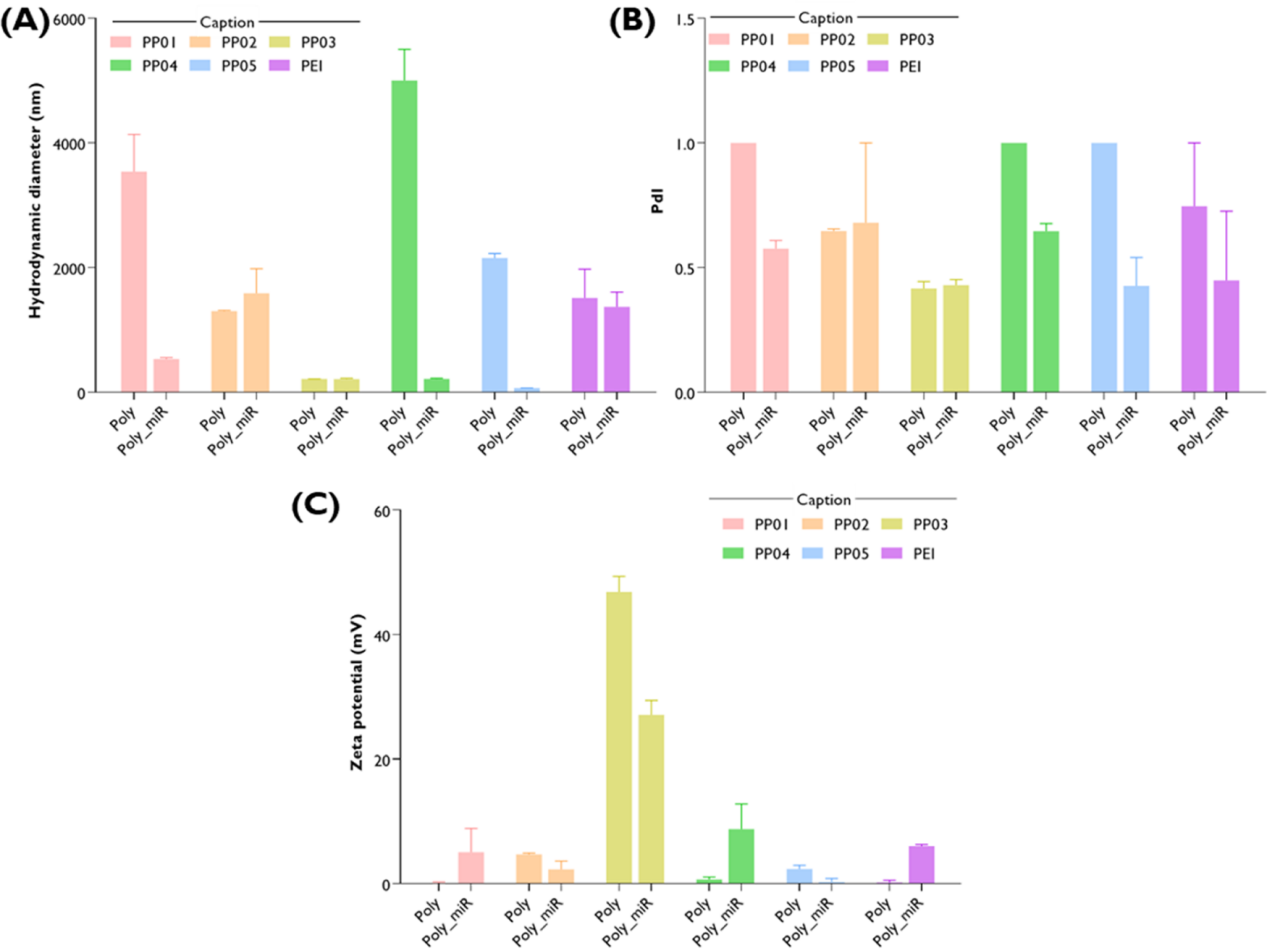
Colloidal properties of the Pluronic–PEI conjugates (PP01 to PP05) and the native B-PEI (PEI) complexed or not with miRNA100 at an N/P ratio of 5. (A) Hydrodynamic diameter (nm), (B) polydispersity index (PdI), and (C) zeta potential (mV) were determined in HEPES buffer (20 mM, pH 7.4) at 37 °C. Poly = polymer; Poly_miR =polymer complexed with miRNA100 at an N/P ratio of 5.

Regarding the zeta potential evaluation, it was observed that the complexation with miRNA increased the surface charge of PP01, PP04, and native B-PEI-based polyplexes, suggesting a rearrangement of polymer chains that exposes additional positively charged groups.^78^ In contrast, PP02, PP03, and PP05-based polyplexes showed a decrease in zeta potential, indicating that miRNA may partially shield the cationic polymer surface. Alternatively, in the case of PP03, the charge decreases from approximately +40 mV to +30 mV, which may indicate that miRNA loading is not restricted to surface complexation but can also penetrate the nanogels’ pores during encapsulation.^77^

### 3.4 Mucoadhesion Studies and Physical Parameters

Since in situ oral cancer is localized in the oromucosal region and could be a route of particular interest for delivery of therapeutic cargos, the mucoadhesive properties of the synthesized polymers were assessed (Figure 7). Therefore, a turbidimetric assay that allows assessment of the interactions between mucin and synthesized polymers was used (Figure 7A,B). It was possible to clearly observe that PP03 outperforms the other synthesized polymers when mucin is prepared in ultrapurified water (Figure 7A) or in nonenzymatic artificial saliva (Figure 7B). In water, PP03 and LMW-chitosan, a recognized mucoadhesive polymer used as a positive control, presented similar interaction profiles with mucin.^42^ The onset of strong interactions was observed at low polymer/mucin ratios (*w*/*w* 0.1) decreasing rapidly with mucin increase, as was observed for other positively charged polymers indicating prevalence of electrostatic interactions.^43^ On the contrary, in saliva, interactions of PP03 and mucin progressively increase with the increase of polymer/mucin (*w*/*w*) ratio, suggesting the growing contribution of nonelectrostatic interactions between the polymers and mucin.^43^ While LMW-chitosan interactions steeply decrease with increasing amounts of the polymer (*w*/*w* 0.5–0.6), growing concentrations of PP03 result in increasing interactions with mucin. The other synthesized polymers (PP01, PP02, PP04, and PP05) as well as the native B-PEI appeared to not interact with mucin presenting similar profile as the negative control, LMW-HA. To confirm these results, adhesive properties were analyzed by using a texturometer apparatus coupled with a mucoadhesion probe (Figure 7C). The results revealed that PP03 present mucoadhesive properties which was translated into the increase in the work of adhesion and the detachment force. In water (Figure 7D), statistical differences were observed when compared to LMW-chitosan in the detachment force, which was less than 1 N for LMW-chitosan and nearly 2 N for PP03. Moreover, in the presence of nonenzymatic artificial saliva (Figure 7E), like in the turbidimetry assay, stronger interactions occurred between PP03 compared to LMW-chitosan. A statistically significant increase in the work of adhesion (1.5 to 2 N·sec for LMW-chitosan and PP03, respectively), as well as in the detachment force (1.2 for LMW-chitosan and 2 for PP03), was observed. Interactions with mucin also influenced the colloidal properties of the complexes formed with LMW-chitosan and PP03 at different *w/w* ratios (Figure 7F,G). It is particularly interesting that in the case of PP03, the size reduces at *w*/*w* 0.25/1 (polymer/mucin) to the values observed for the free polymer (ca. 200 nm), suggesting that mucin could intercalate into the polymeric network. In comparison, the size of complexes between LMW-chitosan and mucin remains in the micrometer region (Figure 7F). Interestingly, the zeta potential of the complexes increased as the polymer/mucin (*w*/*w*) increases and vice versa denoting that the negatively charged mucin (ca. −5 mV) is interacting with the positively charged polymers (LMW-Ch ca. 70 mV and PP03 ca. 50 mV) (Figure 7G). In summary, these results revealed that PP03 exhibits outstanding mucoadhesive properties, making it an attractive vehicle for oromucosal applications.

**Figure 7:**
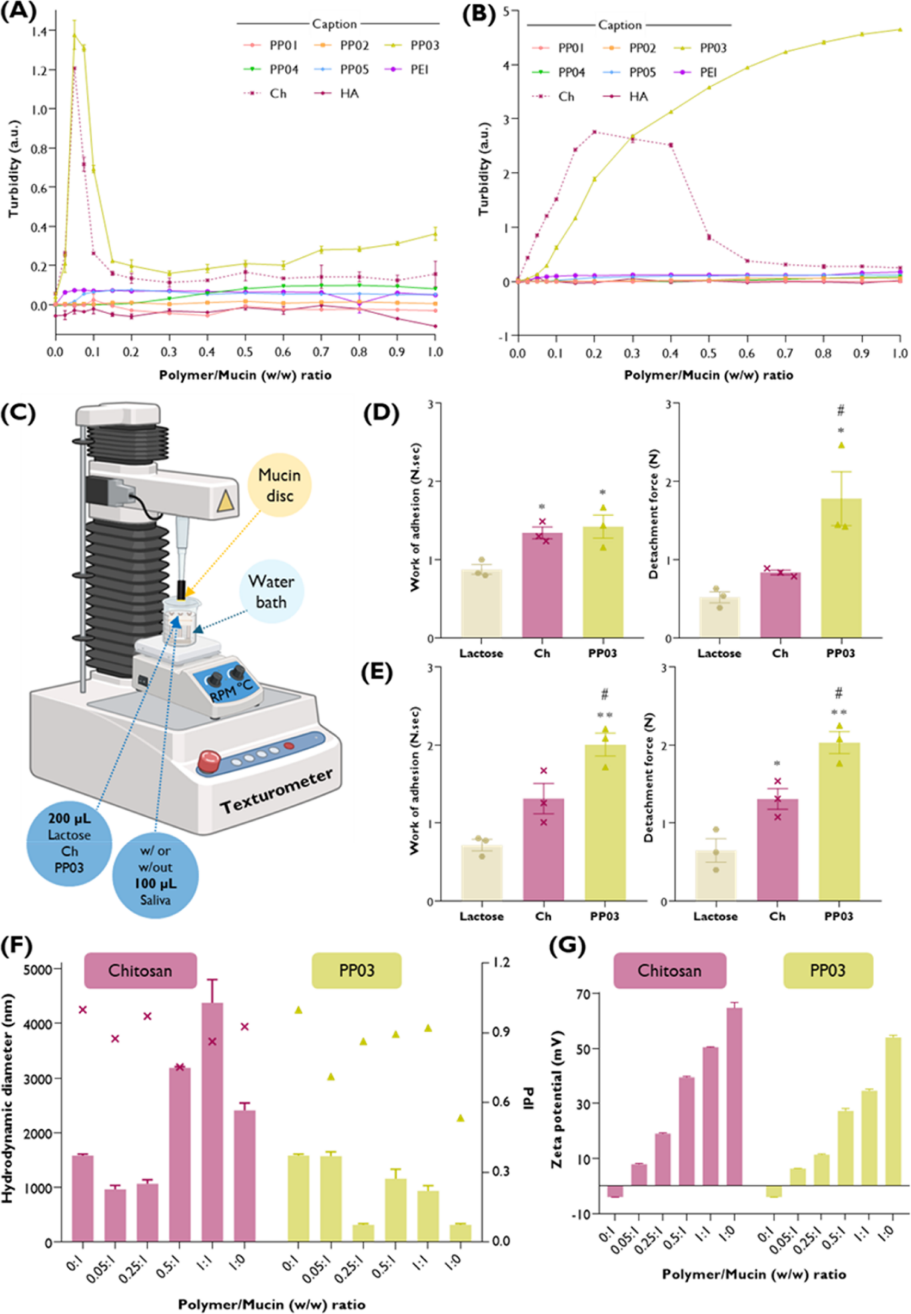
Mucoadhesion studies. Turbidimetric titration of mucin by different synthesized polymers (PP01 to PP05) and native B-PEI (PEI) in different polymer/mucin (*w*/*w*) ratios using (A) ultrapurified water or (B) nonenzymatic artificial saliva as dispersants. LMW-Chitosan (Ch) and LMW-hyaluronic acid (HA) were used as positive and negative controls for mucoadhesion, respectively. (C) Schematic representation of the texture analyzer-based mucoadhesion assay and the respective obtained results of work of adhesion (N·s) and detachment force (N) for PP03, LMW-chitosan (positive control for mucoadhesion) as well as for lactose (negative control for mucoadhesion) prepared in (D) ultrapurified water or (E) nonenzymatic artificial saliva. (F, G) The colloidal properties of the polymers (LMW-chitosan or PP03) and mucin dispersions prepared in different polymer/mucin (w/w) ratios were represented by (F) hydrodynamic diameter (nm) and PdI, as well as (G) zeta potential (mV) measurements. The results represent the mean ± SEM of *n* ≥ 3 independent experiments. Note that some error bars are too small to be shown. Significant differences were defined as follows: *p < 0.05, **p < 0.01 (compared to lactose), and ^#^ *p* < 0.05 (compared to LMW-chitosan).

Considering this, we further assess the thermogelation behavior of PP03. Consequently, the PP03 formulation demonstrated thermogelation properties, forming a gel within one min when 20 μL of the dispersion at 80 mg·mL^–1^ was placed on a plastic square weighing boat at 37 °C, instead in the presence of 10 μL of artificial saliva. This rapid gelation also advantageously contributes to oromucosal administration, as it allows for quick onset of action upon contact with mucosal surfaces.^79^

Given the potential application of PP03 for intravenous delivery of therapeutic cargos, osmolality and sterility are important prerequisites to assess. The osmolality of the optimized PP03 formulation was approximately 183 mOsm/kg, comparable to hypotonic NaCl solutions (0.33–0.45%) commonly used in intravenous medicinal preparations, which may indicate its potential application for parenteral administration.^80^ Microbiological assessment demonstrated no detectable microbial growth under the tested conditions, confirming the formulation’s compliance with sterility standards and its suitability for parenteral administration, as recommended in the ICH Q6A guidelines.

### 3.5 Impact of Synthesized Polymers on Oral Cancer Cells and Their Hemolytic Potential

The biological activity of the different native Pluronics (Figure S6) and B-PEI (1.8 kDa), as well as the synthesized polymers (Figure 8A,B), was evaluated in 2D in vitro cell models of oral cancer.

**Figure 8:**
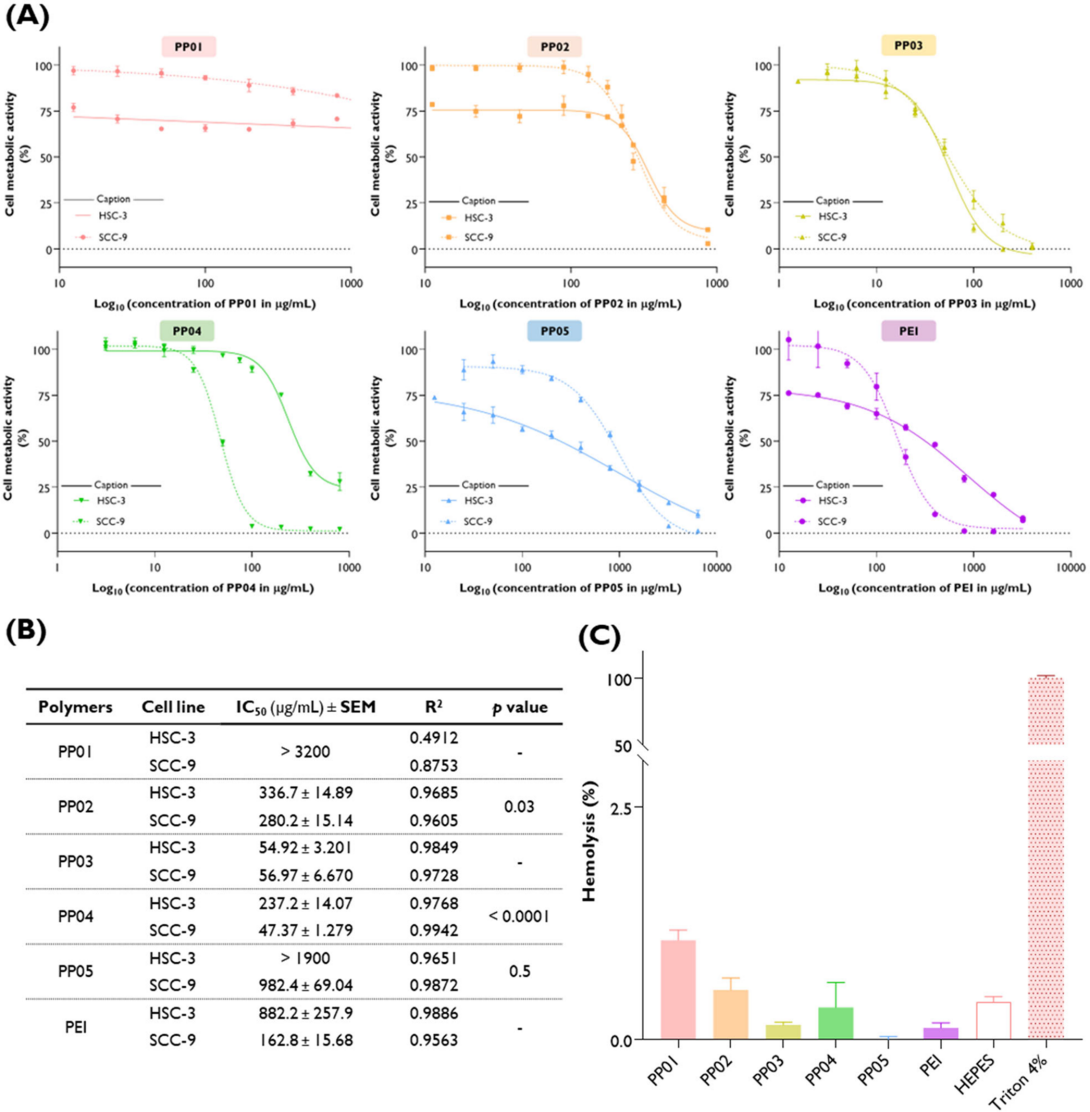
Effect of Pluronic–PEI conjugates (PP01–PP05) and native B-PEI (PEI) on the metabolic activity of SCC-9 and HSC-3 cells and their hemolytic potential. (A) Cell metabolic activity in SCC-9 and HSC-3 cells was assessed 48 h post-incubation with increasing concentrations of the different polymers using the resazurin assay. (B) Half-maximal inhibitory concentrations (IC_50_) were determined via non-linear sigmoidal curve fitting, with R^2^ values confirming good model fit and p-values indicating significant differences between the two cell lines. (C) Percentage (%) of hemolysis produced by the different polymer dispersions after 1 h of incubation. 0.9% NaCl and 4% Triton X-100 were used as negative and positive controls, respectively. Polymer dispersions were prepared in HEPES buffer (20 mM, pH 7.4). The results represent the mean ± SEM of *n* ≥ 3 independent experiments. Note that some error bars are too small to be shown.

As shown in Figure S6, the exposure of HSC-3 and SCC-9 cells to increasing concentrations of unmodified Pluronic has demonstrated a reduction in cell metabolic activity. The tested Pluronics and their dose influenced these results, which depend on the specific cell line being tested. These findings are in line with previous reports by Kabanov and colleagues, who demonstrated that certain Pluronic block copolymers can modulate cellular bioenergetics, membrane fluidity, and mitochondrial function in a concentration- and cell type-dependent manner.^57,81^ Therefore, these results may open doors to further exploring and verifying the potential application of Pluronics as selective active ingredients in modulating oral carcinogenesis.

Moreover, the synthesized polymers induced dose-, time-, and cell-type-dependent reductions in the metabolic activity of oral cancer cells (Figure 8A). The most pronounced effect (Figure 8B) was recorded for PP03 with a half-maximal inhibitory concentration (IC_50_) of ca. 55 μg·mL^–1^, in both localized (SCC-9) and metastatic (HSC-3) oral cancer cell lines. The high ZP implicates strong interactions with the negatively charged components present on the cell membrane and possible destabilization of cellular membranes which may compromise cellular membrane integrity. However, the lack of hemolytic effect observed for all synthesized polymers (Figure 8C) may suggest tumor cell-specific targets and mechanism of activity. This difference may arise from the distinct membrane compositions and repair mechanisms between erythrocytes and malignant epithelial cells, as well as the steric shielding and amphiphilic architecture provided by Pluronic segments, which mitigate nonspecific interactions with red blood cells, supporting the observed biocompatibility with red blood cells.^82,83,81^

### 3.6 PP03 Outperforms in Vehiculating RNAi and Hydrophobic Probe

To understand which synthesized polymer could be a better vehicle for miRNA into cancer cells, the cellular uptake of the different polymers complexed with siRNA-Cy5 was assessed by flow cytometry (Figure 9). The results revealed that after 4 h of incubation with polyplexes, a statistically significant increase in the mean intensity of fluorescence was observed in the cells treated with PP03-siRNACy5, demonstrating that this cross-linked polymer increases the uptake of the siRNA compared with all the others, including the B-PEI. These results are evident for the two OSCC cell lines in the study, SCC-9 (Figure 9A) and HSC-3 (Figure 9B). However, the uptake studies revealed cell-dependent uptake, indicating selective uptake by the invasive cell type (HSC-3) (Figure 9C).

**Figure 9:**
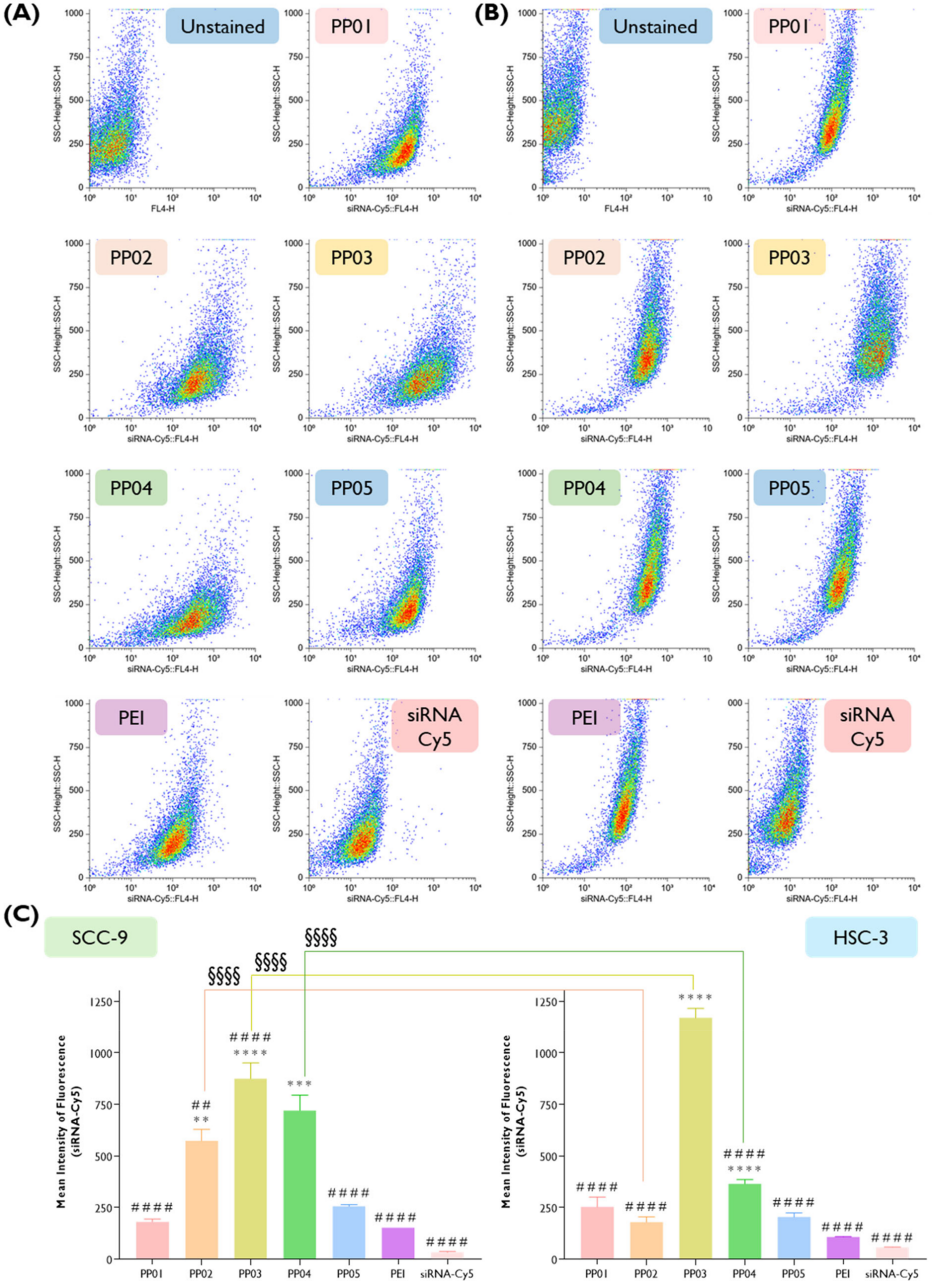
Cellular uptake of the polyplexes formed by the different synthesized polymers and the siRNA-Cy5. Representative dot plots of (A) SCC-9 and (B) HSC-3 cells: untreated, treated with scrambled siRNA-Cy5 complexed with synthesized polymers (PP01–PP05) or native B-PEI (PEI), and treated with scrambled siRNA-Cy5 without a transfection agent. (C) The quantitative mean fluorescence intensity of the siRNA-Cy5 in the SCC-9 and HSC-3 cells, respectively. The results represent the mean ± SEM of *n* ≥ 3 independent experiments. Significant differences were defined as follows: **p < 0.01, ***p < 0.001, and ****p < 0.0001 (compared to B-PEI, labeled as PEI in the graph); ^##^ *p* < 0.01, and ^####^ *p* < 0.0001 (compared to PP03); as well as ^§§§^ *p* < 0.001, representing differences in polyplex performance between SCC-9 and HSC-3 cells.

Considering the presence of hydrophobic components in the PP03 network, the ability of this polymer to transport hydrophobic cargos was also evaluated, using the hydrophobic probe C6 (Figure 10). As is possible to observe, PP03 increased the uptake of C6 more than 6-fold compared to the free probe (Figure 10A), which was also evident in the fluorescence microscopy (Figure 10B).

**Figure 10:**
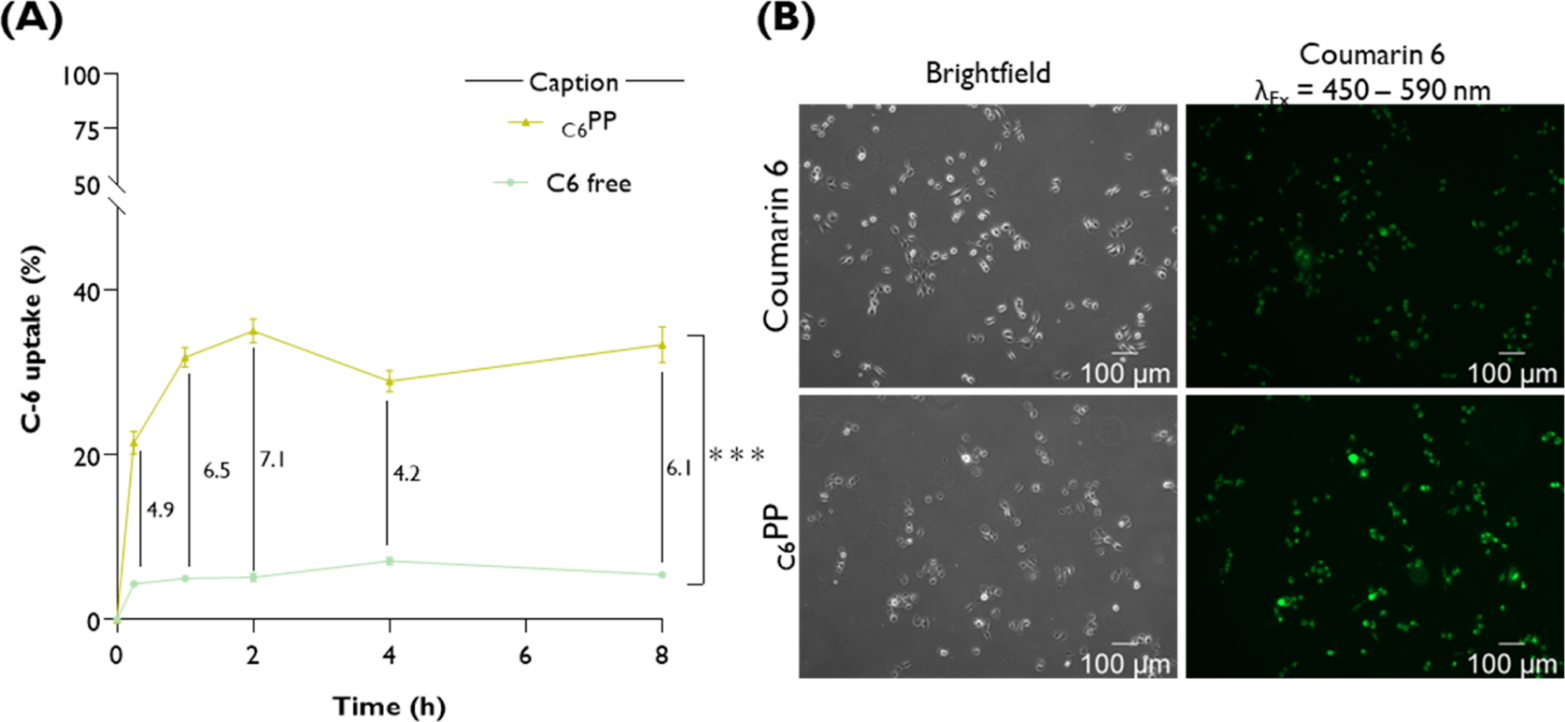
Hydrophobic Coumarin 6 (C6) dye uptake profiling in HSC-3 cells in its free form (C6 free) or encapsulated in PP03 (C6PP). (A) C6 was quantified by fluorescence spectroscopy in HSC-3 cells, and the results were plotted as C6 uptake (%) registered over 8 h at 37 °C. Data were expressed as mean ± SEM of n ≥ 3 independent experiments. Significant differences were defined as follows: ***p <0.001 (C6 free vs _C6_PP at the end of the experiment). Note that some error bars are too small to be shown. (B) Representative live confocal microscopy photographs obtained with a 10× objective. Scale bar: 100 μm.

Although PP03 can be effective in delivering both types of molecules (RNAi and hydrophobic probes), their combined delivery and therapeutic implications still need to be determined and validated in future experiments.

### 3.7 Morphology of Cross-Linked PP03 Indicates the Micellar–Nanogel Architecture

Particle shape and surface roughness can work synergistically to influence the formation and stability of particle networks in colloidal gels.^84^ Moreover, the design and structure–activity may also be implicated in the performance of delivering RNAi cargos.^85^ Together, they participate in the in vivo transport of the nanoparticles.^85^ The morphological features of PP03 demonstrated the presence of an amorphous-like structure composed of small spheres inside the microstructure (Figures 11A and S6), indicating a spherocolloidal arrangement in which the polymer network is reinforced by or incorporates spherocolloidal particles (Figures 11B and S7B), with compatible 3D-like feature characteristics of an amorphous polymer surface with some roughness, which may confer more stability, limiting the sedimentation of the particles^84^ (Figure 11C). Furthermore, TEM analysis also revealed a nanogel architecture composed of spherical nanoparticles interconnected in a cross-linked-like configuration at concentrations of ca. 20 mg·mL^–1^ (Figure S8) and 50 μg·mL^–1^ (Figure 11D). At 50 μg·mL^–1^, the nanoparticles within the gel network exhibited a dense core with an average diameter of 119.9 ± 5.9 nm. This micellar–nanogel structure enables dilution below the CMC, determined to be 68 μg·mL^–1^ at 25 °C and 38 μg·mL^–1^ at 37 °C (Figure S9). Remarkably, the formulation remained stable even below 25 μg·mL^–1^, displaying well-defined spherical nanoparticles with a mean core diameter of 84.3 ± 8.0 nm (Figure 11E).

**Figure 11:**
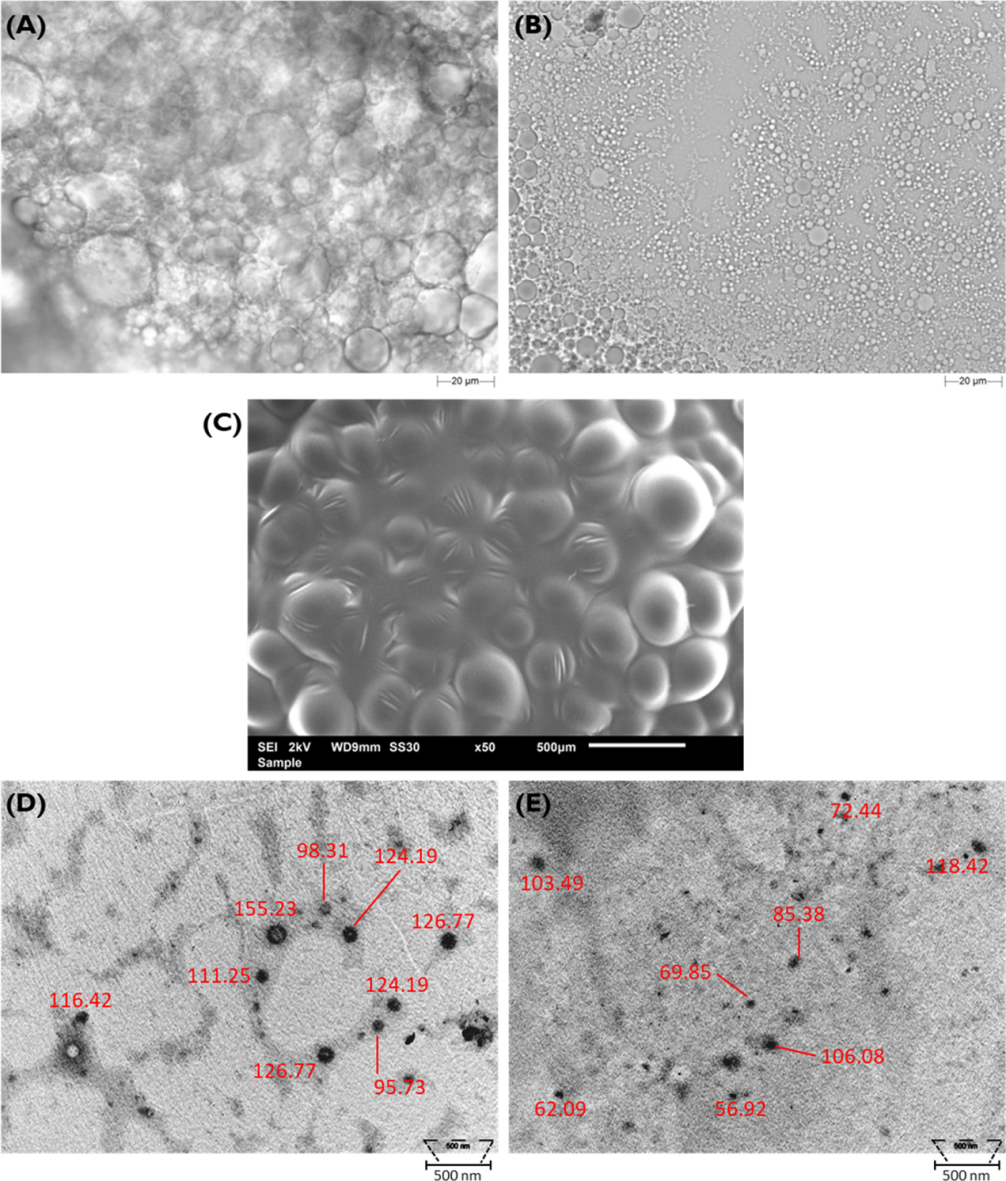
Morphological and structural arrangements of PP03. In bulk, PP03 presents a (A) cotton-like surface, typically observed in amorphous polymers, and a (B) microstructure with characteristics of a spheroidal hydrogel. Scale bar: 20 μm. (C) The surface PP03 aspect demonstrates a 3D rough profiling. Scale bar: 500 μm. PP03 architecture prepared at a concentration of (D) 50 μg·mL^–1^ and (E) 25 μg·mL^–1^, showing evidence of micelles inside a networking gel. Scale bar: 500 nm. In red, the size (nm) of the dense core of the particles that are present in the interconnective network.

The smaller particle sizes observed in TEM, relative to DLS measurements, are likely due to dehydration-induced collapse of hydrated coronas and solvation layers during sample preparation,^33^ which may also be challenging in observing micelle packing within the hydrated gel phase. Therefore, future studies employing Cryo-TEM and/or small-angle X-ray scattering (SAXS) will be of interest to resolve the nanoscale organization and internal structural arrangement of micelles within the gel matrix, under conditions that better preserve native morphology.^86^

### 3.8 PP03 as a Micellar Nanogel to Vehiculate Therapeutic miRNA 100 in 2D and 3D Models of Oral Cancer Cells

Polyplexes composed of PP03 and scrambled or therapeutic miRNA100 were formed at *N*/*P* 5 by an optimized protocol depicted in Figure 12A. The morphological images of PP03miR100, obtained by TEM, showed spherical polyplexes embedded within a gel-like matrix with sizes of ca. 116.4 ± 5.7 nm (Figure 12C), resembling those of the empty PP03 matrix (Figure 11E). The PP03miR100 polyplexes significantly reduced the metabolic activity of HSC-3 cells compared to those formed by scrambled miRNA or B-PEI-derived polyplexes (Figure 12B). Importantly, empty PP03 decreased the metabolic activity of normal tongue epithelium by about 30%, which is within the threshold of international guidelines (ISO 10993-5:2009­(E)) and represents a concentration 7 times higher than the one used for therapeutic schemes (Figure 12D). Moreover, no hemorrhagic events were caused by empty PP03 at concentrations between 500 and 1000 μg·mL^–1^ (Figure 12E). Additionally, advantageously, PP03-derived polyplexes formed with either scrambled miRNA or miRNA100 did not induce hemolysis (Figure 12F), as all tested samples showed hemolysis levels below 2% (ASTM F756-00 standard), indicating favorable hemocompatibility and supporting their safety profile for potential intravenous administration.

**Figure 12:**
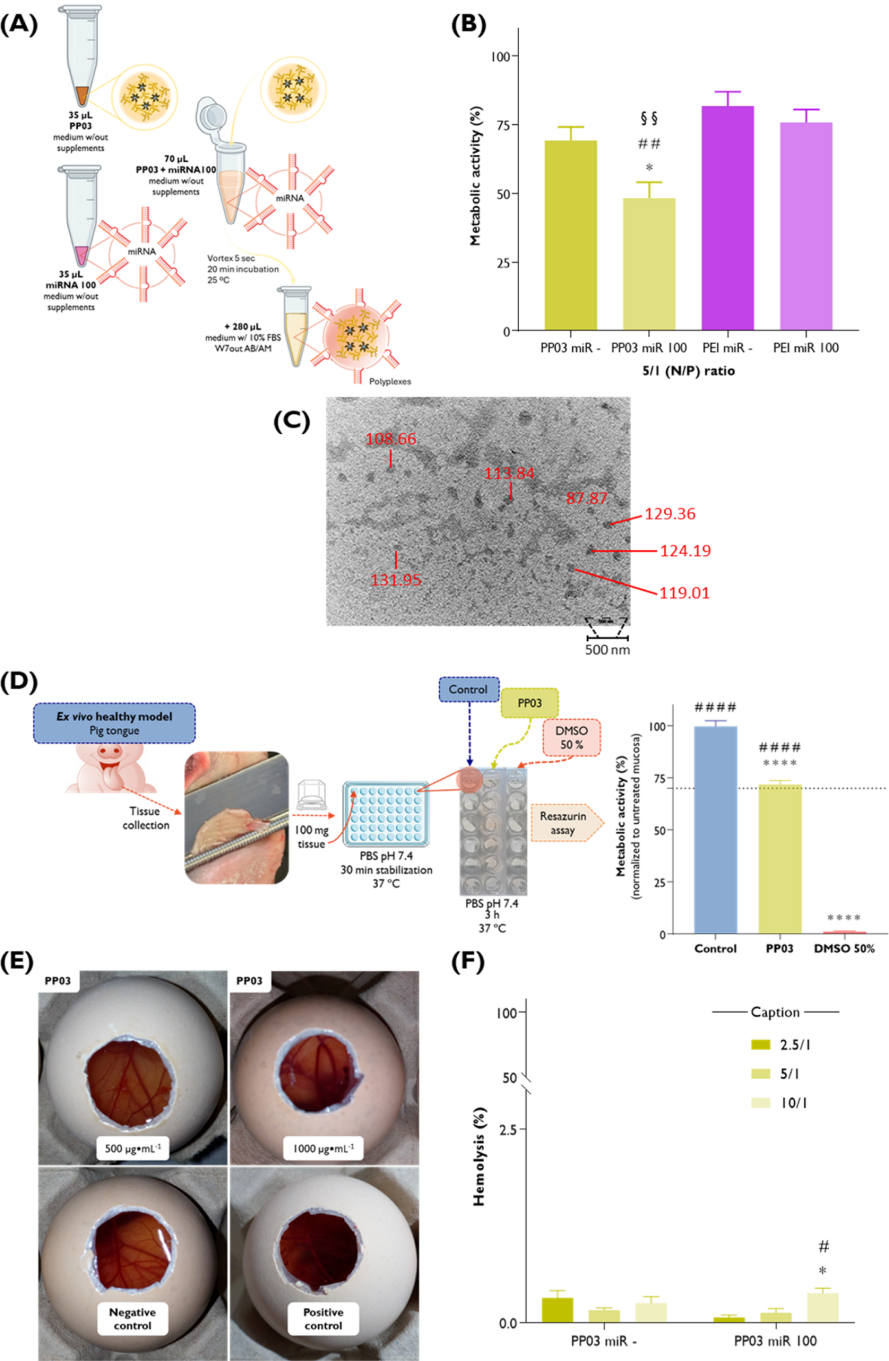
Preparation and evaluation of PP03 and B-PEI polyplexes with scrambled miRNA (miR −) or therapeutic miRNA100 (miR100) at an N/P ratio of 5 in HSC-3 oral cancer cells, including assessment of their effects on normal tissues. (A) Schematic representation of the polyplexes preparation. (B) The PP03miR100 polyplexes significantly reduced the metabolic activity of HSC-3 cells compared to PP03miRNA- and PEI polyplexes. Significant differences were defined as follows: *p < 0.05 (PP03miR100 vs PP03miR-); ^##^ *p* < 0.01 (PP03miR100 vs PEImiR-): as well as ^§§^ *p* < 0.01 (PP03miR 100 vs PEImiR100). (C) Morphology of PP03miR100 at a N/P ratio of 5 (80 nM miRNA) obtained by TEM. Scale bar: 500 nm. In red, the size (nm) of the dense core of the particles. (D) Ex vivo tongue epithelium preparation to assess the impact of PP03 on its metabolic activity. Dimethyl sulfoxide (DMSO, 50%) was used as a control for the reduction of cell metabolic activity. Significant differences were defined as follows: ****p < 0.0001 (compared to control, i.e., untreated mucosa); and ****p < 0.0001 (compared to DMSO 50%). (E) Photographs of the HET-CAM results after the exposure to PP03 (500 and 1000 μg·mL-1). 0.9 % NaCl and 1M NaOH were used as positive and negative controls, respectively. (F) Percentage (%) of hemolysis produced by PP03miR- and PP03miR100 at increasing N/P ratios (2.5 to 10) after 1 h of incubation. 0.9% NaCl and 4% Triton X-100 were used as negative and positive controls, respectively. The results represent the mean ± SEM of *n* ≥ 3 independent experiments. Significant differences were defined as follows: *p < 0.05 (PP03miR 100 at N/P ratio of 10 vs N/P ratio of 2.5) and ^#^ *p* < 0.05 (PP03miR 100 at N/P ratio of 10 vs N/P ratio of 5).

The observed therapeutic effects of miRNA100 may indicate the ability of PP03 to colocalize its cargo within the cytoplasm, suggesting either the prevalence of the endocytic mechanism that bypasses the endosomal system or the ability of PP03 to provide successful endosomal escape. Therefore, to better elucidate the endolysosomal escape of PP03miR100 and PEImiR100 polyplexes, the AO assay was employed. AO, a cell-permeable green fluorophore, becomes protonated and accumulates in acidic vesicular organelles (AVOs), forming red fluorescent aggregates. Upon release into the neutral cytosol, AO returns to its green fluorescent monomeric form, providing a direct indication of endolysosomal escape and cytoplasmic delivery of the formulations (Figure 13A).^87,88^ In the kinetic study (Figure 13B), HSC-3 cells exposed to PP03miR100 showed a statistically significant decrease in red-to-green fluorescence of AO over 240 min compared to cells treated with PEImiR100. This observation was corroborated by fluorescence microscopy photographs acquired after 240 min under the same treatment conditions (Figure 13C). These results may indicate that the presence of cationic PEI is not sufficient to induce endosomal escape and colocalization of miRNA within the cytoplasm. Actually, the action mechanism of effective intracellular colocalization and endosomal escape of nucleic acids by PEI-based nanovehicles has traditionally been ascribed to the interaction of the cationic groups with biomembranes. Nonetheless, the structure and MW of PEI as well as PEI modification could have profound implications on cellular uptake mechanism, endosomal escape, and therapeutic outcomes. Previous studies on LMW PEI conjugated with Pluronics indicated that the Pluronic component significantly improves the transfection of both pDNA and short oligonucleotides when compared to free PEI. In addition, it has been reported that the presence of free Pluronics considerably enhances the transfection efficacy of this class of transfection agents.^66,89^ For example, hydrophobic Pluronics can modulate cell membranes in a Pluronic- and cell-dependent manner, decreasing membrane viscosity, acting as transmembrane carriers, or forming pores depending on their aggregation state, thereby influencing the subcellular distribution of therapeutic molecules.^90^ Hence, the presence of Pluronic in the PP03 could support the protection and the intracellular release of the therapeutic cargo, in this case of the miRNA100. Although our obtained results revealed the absence of free Pluronic in PP03 (Table 1), the presence of a degradable ester linker could be the source of free L121.^91^ Actually, the disappearance of the ester peak near δ 3.9 ppm in the ^1^H NMR spectra demonstrates that PP03 suffers hydrolysis within 48 h of experiment at the pH of the endosomal compartment (pH 5) (Figure 13D).^19,92^ Since Pluronic L121 is a hydrophobic Pluronic (HLB 1–7), it might contribute to endosomolysis (Figure 13E) and to the registered endosomal escape evaluated by the AO assay, ultimately translated into a significant reduction in the metabolic activity of HSC-3 cells treated with PP03miRNA100.

**Figure 13:**
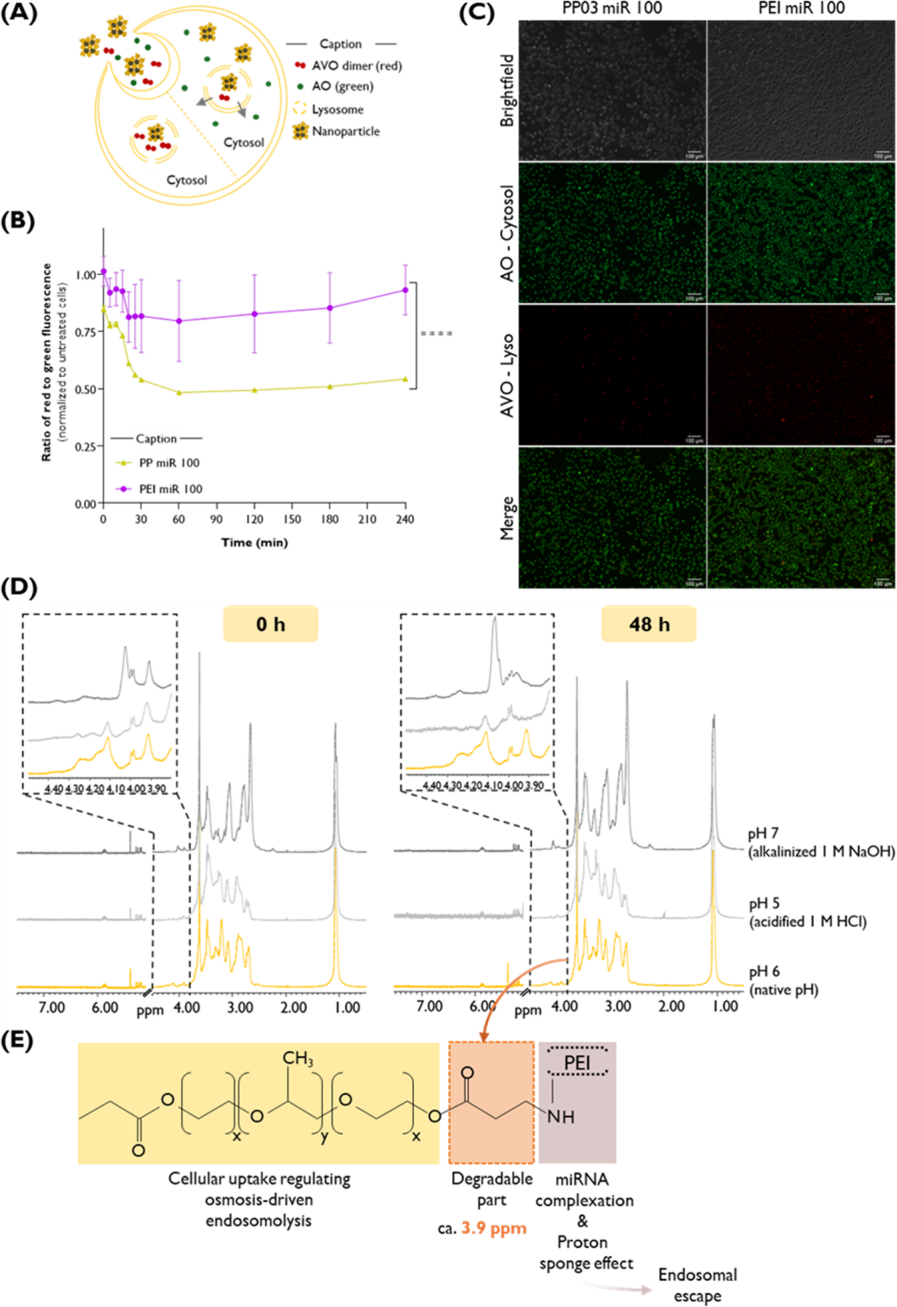
Endosomal escape drives the efficacy of PP03miRNA100 treatment in HSC-3 oral cancer cells. (A) Schematic illustration of acridine orange (AO) labeling used to monitor endosomal escape. AO accumulates in acidic vesicular organelles (AVOs), emitting red fluorescence, whereas its release into the neutral cytoplasm restores green fluorescence, thereby indicating the escape of polyplex formulations from endosomes. (B) Kinetic monitoring of AO red/green fluorescence ratio in HSC-3 cells over 240 min, before and after treatment with PP03miRNA100 (PP miR100) or PEImiRNA100 (PEImiR100), normalized to untreated cells. The results represent the mean ± SEM of *n* ≥ 3 independent experiments. Significant differences were defined as follows: ****p < 0.0001 (PP03miRNA100 vs PEImiR100). (C) Representative live confocal microscopy photographs obtained with a 10× objective. Scale bar: 100 μm. (D) 1H NMR spectra of PP03 dispersions prepared in different pH 6, 5, and 7, at time 0 and after 48 h of incubation at 37 °C. (E) Simplified representation of the molecular backbone of synthesized PP03, highlighting the pH-sensitive degradable ester bond.

The presented results, particularly the presence of acid-labile ester bonds in the PP03 backbone, confirmed by ^1^H NMR, together with functional evidence from AO dequenching and confocal microscopy assays, adequately support the occurrence of endolysosomal escape. Hence, based on this and in the literature,^93^ we have prepared a scheme of the potential mechanism of endosomal escape of PP03 and the consequent release of the miRNA100 into the cytoplasm of the HSC-3 cells (Figure 14). However, to further substantiate our findings and comprehensively elucidate the intracellular trafficking and endosomal escape mechanisms, future studies employing live-cell imaging and colocalization with specific endosomal and lysosomal markers (e.g., Rab5 and LAMP1) could be of advantage in providing deeper mechanistic insights.^13,94^

**Figure 14:**
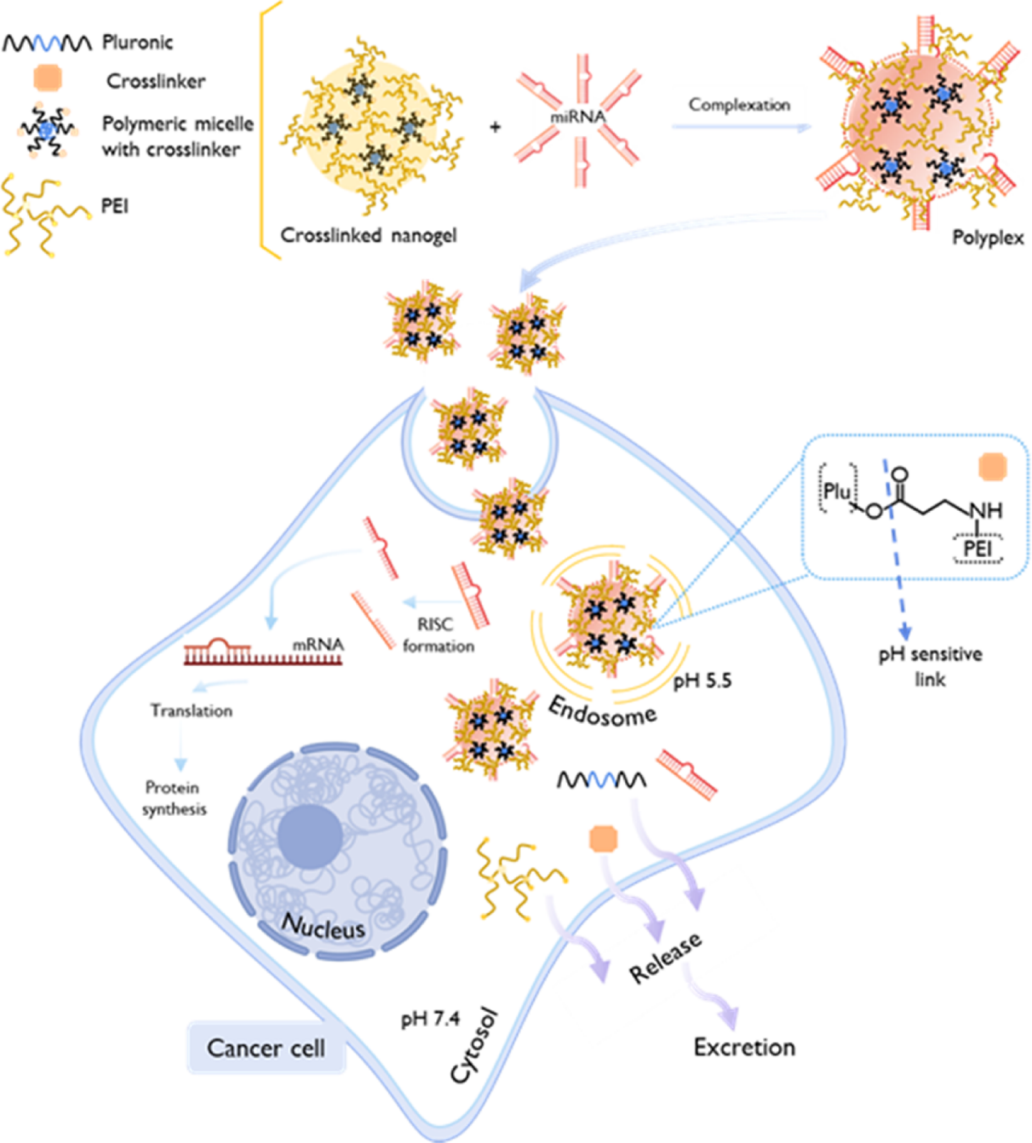
Illustration of the proposed mechanism of PP03-mediated endosomal escape, cytoplasmic release of miRNA, interaction with target mRNA, and potential degradation and excretion of polymer and crosslinker constituents.

To further validate the lead candidate PP03, we employed a homotypic 3D tumor spheroid experiment based on HSC-3 cells, as depicted in Figure 15A.^52^ Compared to traditional monolayer cultures, this model better replicates the tumor microenvironment, including cell–cell and matrix interactions, diffusion barriers, and spatial organization. These features are crucial for evaluating nanocarrier penetration and therapeutic performance in a more realistic setting.^95^

**Figure 15:**
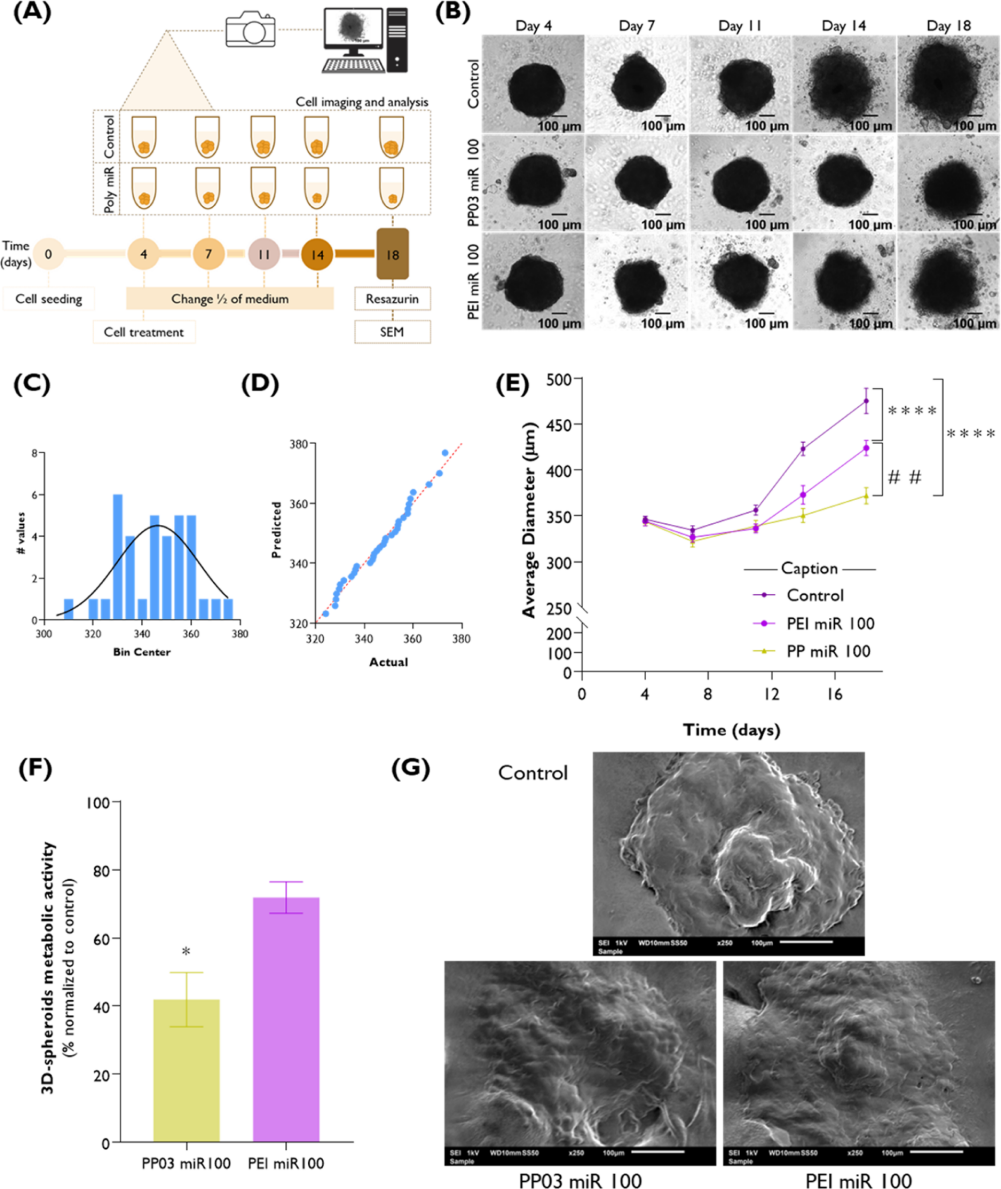
Human HSC-3 tumor 3D-spheroid formation and characterization over 18 days before and after the treatment with PP03miRNA100 (PP03miR100) and PEImiRNA100 (PEImiR100). (A) Schematic chronogram of spheroid development and therapeutic regimen. (B) Representative brightfield microscopy photographs (5× objective) of untreated and treated spheroids (PP03miR100 or PEImiR100) acquired on day 4, 7, 11, 14, and 18, respectively. Scale bar: 100 μm. To guarantee that the diameter of the HSC-3 3D-spheroids on day 4, i.e., prior to treatment, followed a normal distribution, a (C) frequency plotting and a (D) quantile-quantile (Q–Q) plot were generated. (E) Growth kinetic curves of the different spheroid groups, represented as the average diameter (μm) measured over 18 days. The results represent the mean ± SEM of *n* ≥ 3 different spheroids per group. Significant differences were defined as follows: ****p < 0.0001 (compared to the control, i.e., untreated spheroids) and ^##^ *p* < 0.01 (PP03miR100 vs PEImiR100). On day 14, after acquiring brightfield microscopy photographs, 50% of the medium was replaced with the resazurin-containing medium, and the (G) metabolic activity of the spheroids was assessed. The results represent the mean ± SEM of *n* ≥ 3 different spheroids per group. Significant differences were defined as follows: *p < 0.05 (PP03miR100 vs PEImiR100). After, (H) representative photographs of the 3D structure of the control and the treated spheroids (PP03miR100 or PEImiR100) were taken using Scanning Electron Microscopy (250× magnification). Scale bar: 100 μm.

Before starting the treatment regimen, we have ensured that all the spheroids follow a standard size distribution (Figure 15C,D). The results revealed that PP03miRNA100 significantly decreased the tumor spheroid size compared to PEImiRNA100 and the 3D-tumor spheroid control group over the 18 days of the experiment (Figure 15B,E). Moreover, PP03miRNA100 outperformed PEImiRNA100, significantly reducing the cell metabolic activity of the HSC-3 homotypic spheroids at the end of the experiment, day 18 (Figure 15F). At the same time, changes in the morphological features of the outer shell of the sphenoid, compatible with blebbing, were observed, which may indicate possible cell death by apoptosis (Figure 15G), similar to those we have observed in our previous work.^52^

These results highlight the distinct advantages of the PP03 architecture in facilitating efficient and biocompatible RNAi delivery, particularly the therapeutic miRNA100, offering a notable improvement over the native PEI-based system. The enhanced performance observed in a 3D HSC-3 spheroid model, which is more representative of the tumor microenvironment, further supports its translational potential. However, despite the added physiological relevance of 3D cultures, they do not fully mimic systemic biological processes such as immune recognition, organ-specific distribution, and clearance mechanisms. Therefore, to advance the translational potential of the optimized nanocomplexes (PP03miRNA100), follow-up in vivo studies will be essential to validate their therapeutic performance under physiologically relevant conditions. These investigations, including those conducted in oral cancer models previously developed by us,^96^ will provide critical insights into the pharmacokinetics, therapeutic efficacy, and safety profile of the developed formulations.

## 4 Conclusions

The optimization of PEI-based formulations, through strategies such as amphiphilic copolymerization and cross-linking with biodegradable moieties, has shown significant improvements in transfection efficiency. Notably, Pluronic–PEI conjugates enhance the delivery of therapeutic nucleic acids, with the addition of pure Pluronics further boosting transfection efficacy and therapeutic activity.^97,98^ Furthermore, it was also discovered that the addition of pure, unconjugated Pluronics to the Pluronic–PEI conjugate can enhance the transfection efficacy and therapeutic activity of nucleic acids.^66,99^

Our results demonstrated the potential of the cross-linked PP03 nanogel to successfully deliver therapeutic miRNA100 to 2D and 3D in vitro models of highly invasive oral cancer. This unique interlinked structure, arising from the combination of an acrylic linker, hydrophobic Pluronic L121, and LMW-B-PEI, directly contributed to the formation of a colloidal system that remained stable upon dilution under physiological conditions. Oral mucosa is an attractive portal for the noninvasive delivery and treatment of oral cancers since it provides the opportunity to bypass systemic toxicity and loss of sensitive therapeutic cargo such as miRNA. The cross-linked hydrogel demonstrated clear advantage in interactions with mucin over other prepared conjugates and control LMW-chitosan, singling it out as a promising mucoadhesive polymer. A distinctly high positive charge enables efficient miRNA complexation but also imparts anticancer biological activity against both cancer cell lines tested in this study (the aggressive HSC-3 and the in situ SCC-9), which can complement the therapeutic activity of miRNA. PP03 exhibited OSCC cell-dependent toxicity with no hemolytic or hemorrhagic effects. Its enhanced miRNA100 delivery in HSC-3 cells may arise from its structural and compositional features, which promote endosomal escape and reduce metabolic activity more effectively than native B-PEI polyplexes in both 2D and 3D models of highly invasive OSCC. Since the same was not observed for the parent B-PEI, at this point, we conjecture that this effect could be ascribed to the presence of the Pluronic component and its possible interactions with the endosomal membrane. Additionally, the presence of degradable ester bonds in PP03 could potentially moderate the organizational level of L121, thereby contributing to the observed effect over time.

In summary, the cross-linked PP03 nanogel represents a promising strategy for targeted, noninvasive delivery of therapeutic miRNA, offering enhanced transfection efficiency, mucoadhesion, and anticancer activity, with the potential to overcome limitations of traditional gene delivery systems in oral cancer treatment.

## Supplementary Material



## Data Availability

The data presented to reproduce the findings in this study are included in the article and in the Supporting Information. The raw data are available upon reasonable request from the corresponding author (A.F.).

## Notes

Heat flow curves; thermoanalytical curves; X-ray analysis; intensity-based particle size distribution curves; cell metabolic activity; morphological results; TEM micrographs; and critical micellar concentration (DOCX)
