# Supplementary material for: Optimizing Pluronic–PEI Nanocarriers for RNAi Delivery in Oral Cancer: From Polymer Synthesis to Functional Screening
Source: Biomacromolecules. 2025 Oct 6;26(11):7484–511. doi: 10.1021/acs.biomac.5c01011 (PMC12818751; doi:10.1021/acs.biomac.5c01011)
Supplement: Supplementary file 1 [file bm5c01011_si_001.docx]

Title: Optimizing Pluronic®–PEI Nanocarriers for RNAi Delivery in Oral Cancer: From Polymer Synthesis to Functional Screening

Authors and Affiliations:

Cátia Domingues^†,‡,§^ (<https://orcid.org/0000-0001-8432-9839>),

Ivana Jarak^†,ɨ^ (<https://orcid.org/0000-0002-0129-4114>)

Jorge Coelho^£^(<https://orcid.org/0000-0001-9351-1704>)

Rui A Carvalho^ε^ (https://orcid.org/[0000-0003-1820-0353](https://orcid.org/0000-0003-1820-0353))

Francisco Veiga^†,‡^ (<https://orcid.org/0000-0002-1041-0068>)

Carla Vitorino^†,ɸ^ (<https://orcid.org/0000-0003-3424-548X>)

Marília Dourado^§^ (<https://orcid.org/0000-0002-5003-4722>)

Ana Figueiras^†,‡,*^ (https://orcid.org/[0000-0001-8170-1113](https://orcid.org/0000-0001-8170-1113))

^†^ Univ Coimbra, Faculty of Pharmacy, 3000-548 Coimbra, Portugal

^‡^ REQUIMTE/LAQV, Drug Development and Technologies Laboratory, Faculty of Pharmacy, University of Coimbra, 3000-548 Coimbra, Portugal

^§^ Institute for Clinical and Biomedical Research (iCBR) area of Environment Genetics and Oncobiology (CIMAGO), Faculty of Medicine, University of Coimbra, 3000-548 Coimbra, Portugal

^£^ Univ Coimbra, CEMMPRE, Department of Chemical Engineering, Rua Sílvio Lima- Pólo II, 3030-290 Coimbra, Portugal

^ε^ Department of Life Sciences, Faculty of Sciences and Technology, 3000-456 University of Coimbra, Coimbra, Portugal

^ɨ^ Instituto de Investigação e Inovação em Saúde, University of Porto, 4000-235 Porto, Portugal

^ɸ^ Coimbra Chemistry Centre, Faculty of Sciences and Technology, University of Coimbra, 3004-535 Coimbra, Portugal

*Corresponding author

Address: Faculty of Pharmacy of the University of Coimbra, Polo III – Health Sciences Polo, Azinhaga de Santa Comba, 3000-548 Coimbra, Portugal

Tel.: +351 239 488 400

E-mail address: rfigueiras@ff.uc.pt

**SUPPORTING INFORMATION**

*Characterization of the synthesized polymers and B-PEI composition*


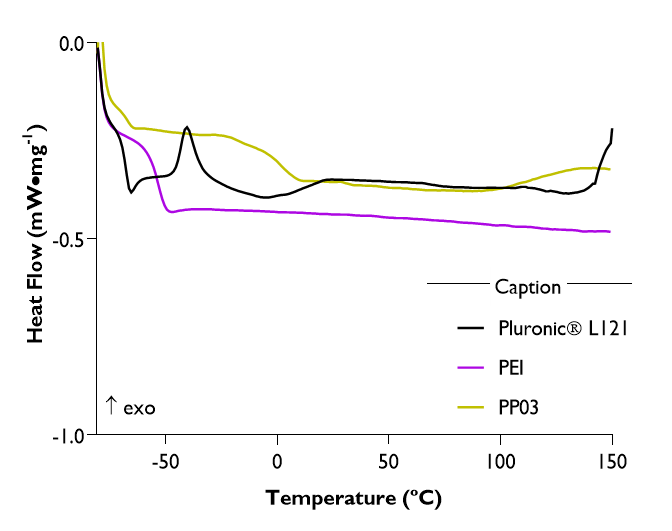


Figure S1 - Heat flow curves of the native Pluronic® L121 and the branched-polyethyleneimine (PEI, 1.8 KDa) as well as the resultant synthesized conjugate PP03, respectively.


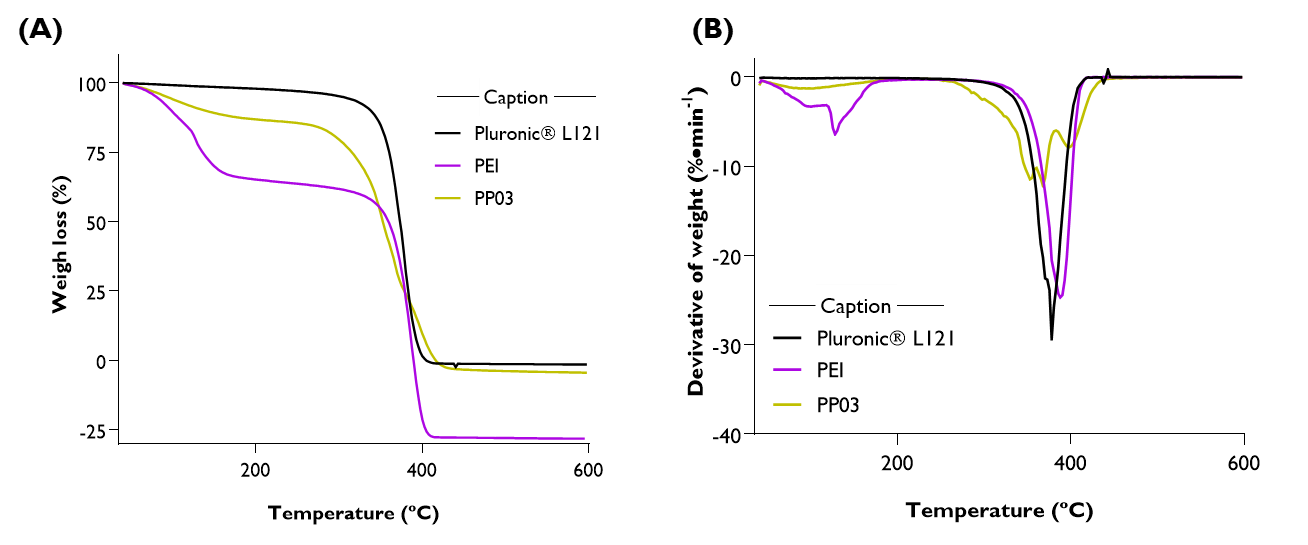


Figure S2 - Thermoanalytical curves of the native Pluronic® L121 and branched-polyethyleneimine (PEI, 1.8 KDa) as well as the resultant synthesized conjugate PP03, respectively. (A) TGA and (B) dTGA.


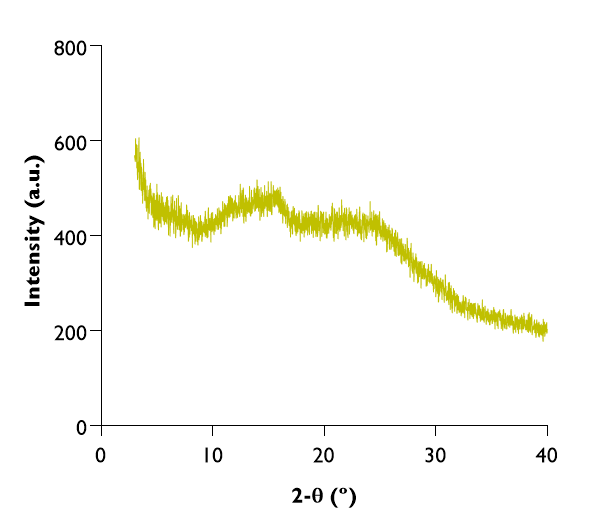


Figure S3 - X-ray analysis of PP03.

*Colloidal properties of synthesized polymers*


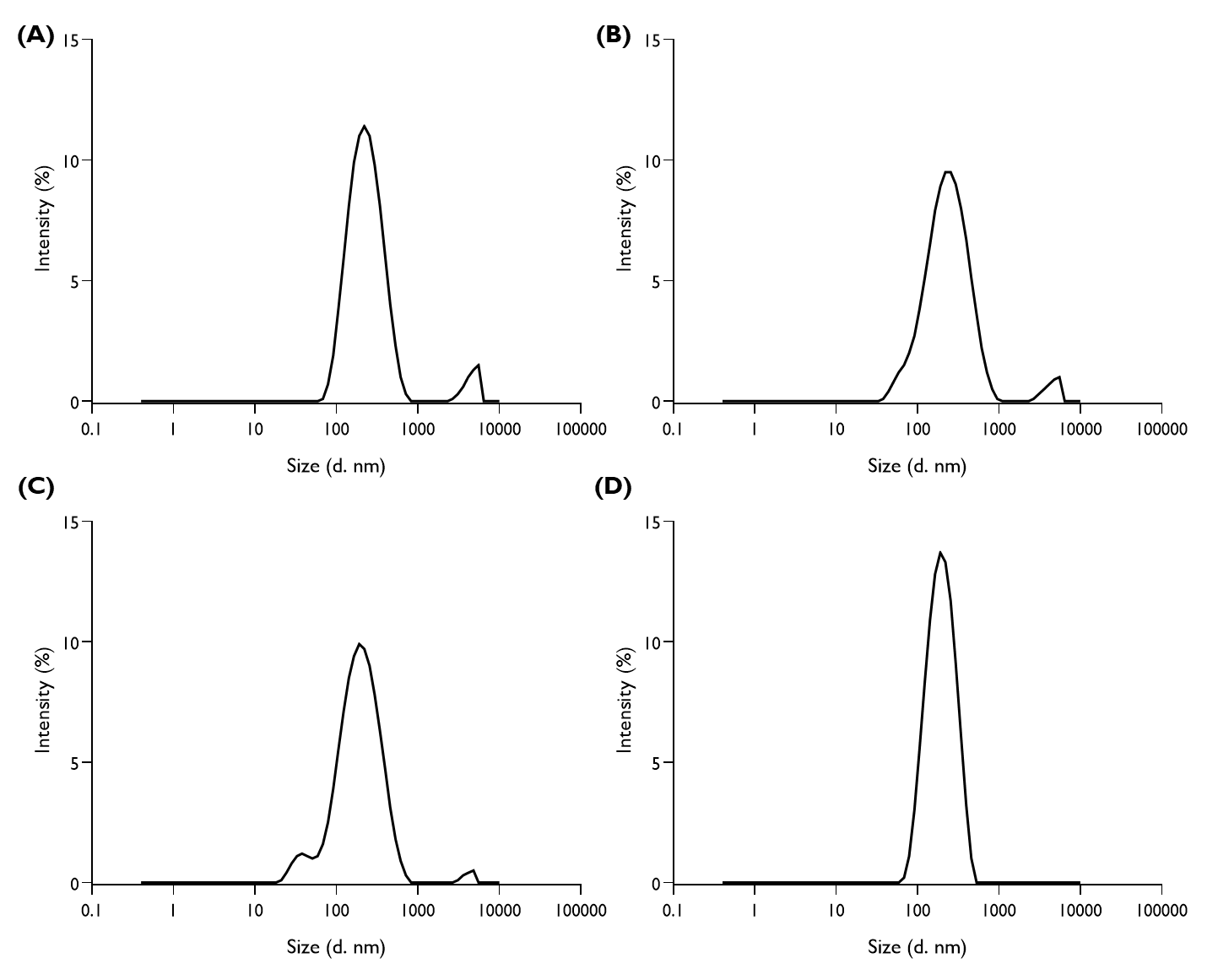


Figure S4 – Intensity-based particle size distribution curves of PP03 in different conditions, obtained through dynamic light scattering (DLS). PP03 was dispersed in (A) nuclease-free water at 10 mg· mL^-1^ or at (B) 1 mg· mL^-1^ at 25 ºC. Moreover, PP03 was also incubated at 37 ºC and prepared at 1 mg· mL^-1^ in (C) nuclease-free water and (D) HEPES 20 mM pH 7.4.

*Buffer capacity, miRNA complexation and stability in serum*

*
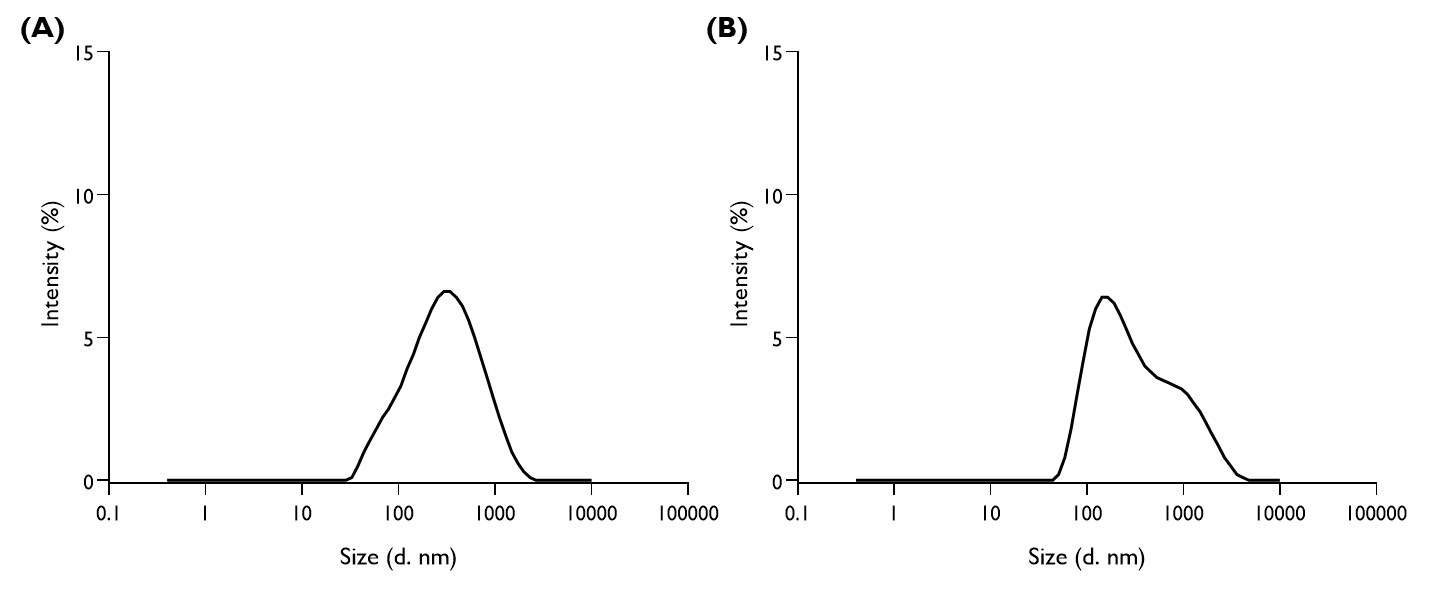
*

Figure S5 - Intensity-based particle size distribution curves obtained through dynamic light scattering (DLS) of PP03 dispersed in HEPES 20 mM, pH 7.4 at 37º (A) before and (B) after complexation with microRNA-100 at a Nitrogen/Phosphate (N/P) ratio of 5.

*The impact of synthesized polymers on oral cancer cells and their hemolytic potential*


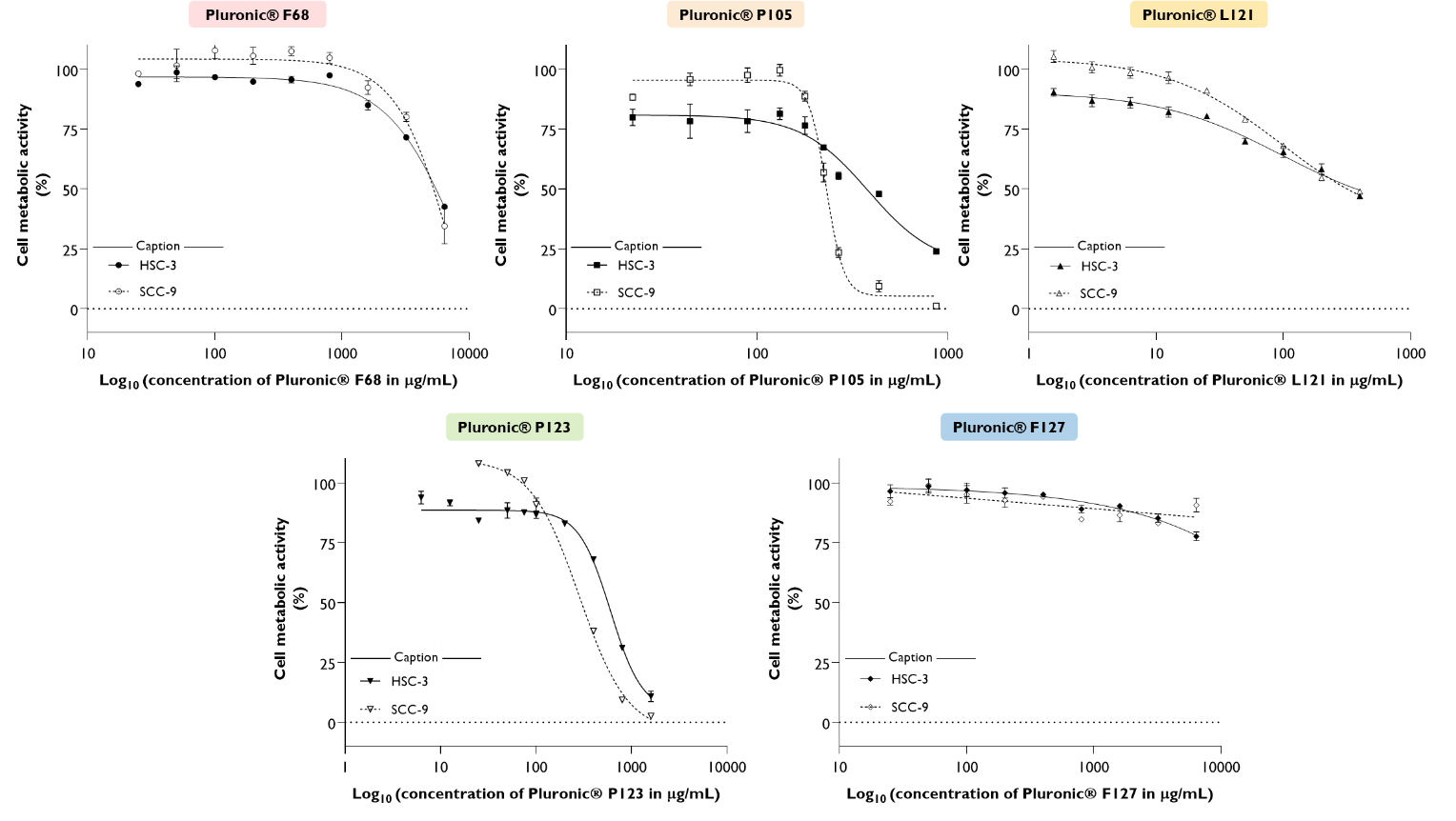


Figure S6 – Cell metabolic activity of SCC-9 and HSC-3 cell lines after 48 h of incubation with increasing concentrations of the different native Pluronics®. Data represent the mean ± SEM of triplicate experiments.

*Morphology of Crosslinked PP03 Indicates Micellar–Nanogel Architecture*


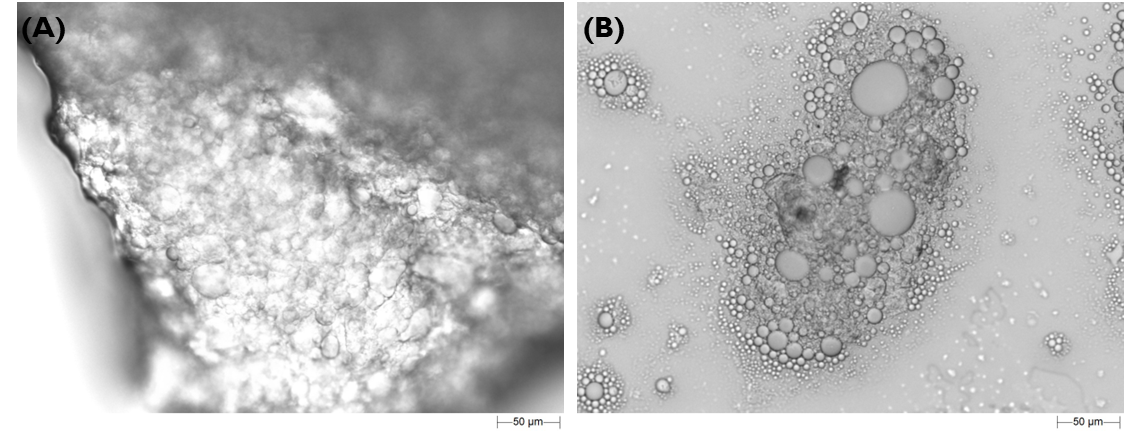


Figure S7 - In bulk, PP03 presents a (A) cotton-like surface, typically observed in amorphous polymers, and a (B) microstructure with characteristics of a spheroidal hydrogel. Morphologi 4 results, scale bar 50 µm.


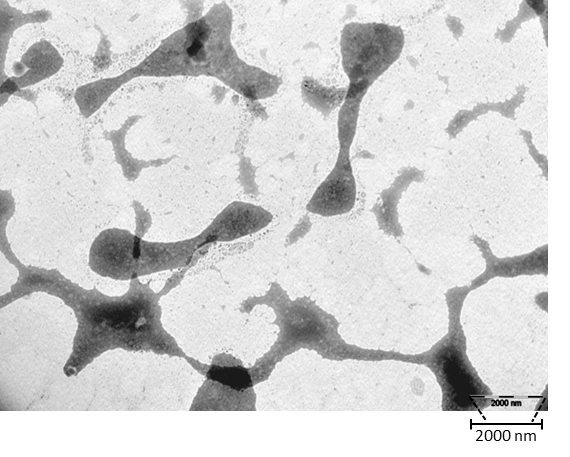


Figure S8 – PP03 at a concentration of ca. 20 mg·mL^-1^. TEM micrographs, scale bar 2000 nm.


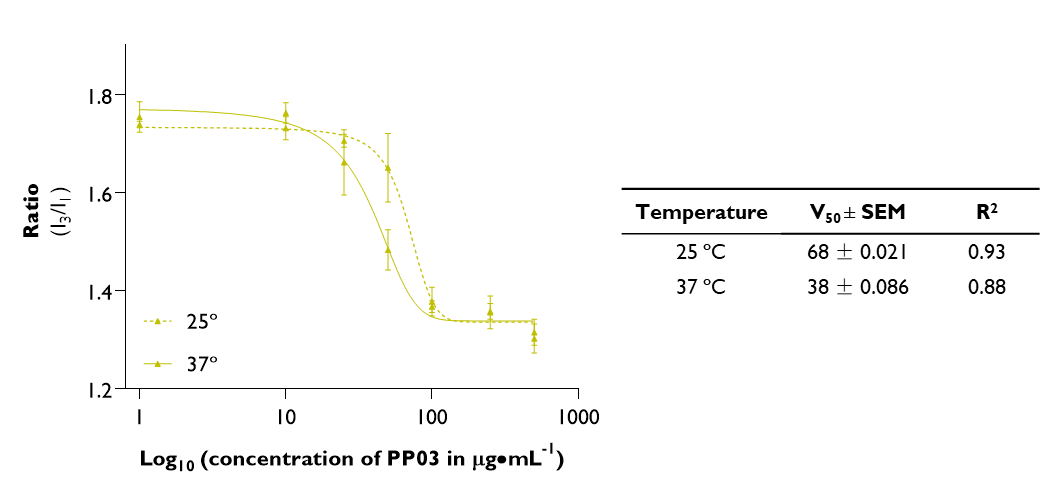


Figure S9 - Critical micellar concentration was assessed by the pyrene assay at two different temperatures, 25 and 37ºC.
